# Super Tough and Spontaneous Water‐Assisted Autonomous Self‐Healing Elastomer for Underwater Wearable Electronics

**DOI:** 10.1002/advs.202102275

**Published:** 2021-09-14

**Authors:** Cyuan‐Lun He, Fang‐Cheng Liang, Loganathan Veeramuthu, Chia‐Jung Cho, Jean‐Sebastien Benas, Yung‐Ru Tzeng, Yen‐Lin Tseng, Wei‐Cheng Chen, Alina Rwei, Chi‐Ching Kuo

**Affiliations:** ^1^ Institute of Organic and Polymeric Materials Research and Development Center of Smart Textile Technology National Taipei University of Technology No. 1, Sec. 3, Chung‐Hsiao East Road Taipei 10608 Taiwan; ^2^ Department of Chemical Engineering Delft University of Technology Delft 2629 HZ Netherlands

**Keywords:** flexible wearable devices, light‐emitting diodes, perovskite quantum dots, underwater electronics, water‐insensitive self‐healing elastomers

## Abstract

Self‐healing soft electronic material composition is crucial to sustain the device long‐term durability. The fabrication of self‐healing soft electronics exposed to high moisture environment is a significant challenge that has yet to be fully achieved. This paper presents the novel concept of a water‐assisted room‐temperature autonomous self‐healing mechanism based on synergistically dynamic covalent Schiff‐based imine bonds with hydrogen bonds. The supramolecular water‐assisted self‐healing polymer (WASHP) films possess rapid self‐healing kinetic behavior and high stretchability due to a reversible dissociation–association process. In comparison with the pristine room‐temperature self‐healing polymer, the WASHP demonstrates favorable mechanical performance at room temperature and a short self‐healing time of 1 h; furthermore, it achieves a tensile strain of 9050%, self‐healing efficiency of 95%, and toughness of 144.2 MJ m^−3^. As a proof of concept, a versatile WASHP‐based light‐emitting touch‐responsive device (WASHP‐LETD) and perovskite quantum dot (PeQD)‐based white LED backlight are designed. The WASHP‐LETD has favorable mechanical deformation performance under pressure, bending, and strain, whereas the WASHP‐PeQDs exhibit outstanding long‐term stability even over a period exceeding one year in a boiling water environment. This paper provides a mechanically robust approach for producing eco‐friendly, economical, and waterproof e‐skin device components.

## Graphical abstract

This novel underwater self‐healing polymer, based on synergistically dynamic covalent Schiff‐based imine bonds with hydrogen bonds, is eco‐friendly, economical, waterproof, and resilient. It has outstanding performance in terms of stretchability (9050%), self‐healing efficiency (95%), self‐healing time (1 h at room temperature), and toughness (144.2 MJ m^−3^), giving it high potential for integration into underwater electronics.

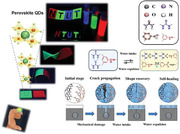

## 1 Introduction

Wearable electronic devices based on skin‐mimetic properties and behaviors have triggered the widespread development of electronic skins (e‐skins).^[^ ^1^, ^2^, ^3^ ^]^ Inspired by the self‐healing core mechanism of biological tissues, which enable the healing and renovation of damaged tissues, self‐healing polymers (SHPs) have been developed to increase the sustainability of e‐skins, reduce their maintenance cost, and explore innovative applications such as flexible electronics, soft robotics, and nanogenerators.^[^ ^4^, ^5^, ^6^, ^7^ ^]^ Studies have demonstrated that skin‐mimetic self‐healing behavior in the fabrication of wearable electronic devices can be achieved through noncovalent bonding—such as hydrogen bonding, metal–ligand interaction, and host–guest interaction—and through covalent bonding through various mechanisms, including Diels–Alder reactions, urea chemistry, olefin metathesis, disulfides, boronic esters, and acylhydrazones.^[^ ^8^, ^9^, ^10^, ^11^ ^]^ Most SHPs are healed through exposure to external stimuli such as light,^[^ ^12^, ^13^ ^]^ heat,^[^ ^14^, ^15^ ^]^ and pressure.^[^ ^16^ ^]^ However, the majority of these self‐healing systems are unstable and sensitive under humid environmental conditions, which limits their long‐term stability in soft electronics. By contrast, water‐induced self‐healing is a promising healing process that occurs at room temperature and is environmentally friendly, economical, and takes environmental protection and human health into consideration. Therefore, the development of a novel eco‐friendly material with waterproof, outstanding long‐term stability, rapid self‐healing efficiency and that can be integrated into wearable electronics remains a considerable challenge.

Water‐based SHPs have been extensively investigated in terms of the facilitation of mechanical damage repair through hydrogen bond interaction dynamics,^[^ ^17^, ^18^, ^19^, ^20^, ^21^, ^22^ ^]^ boronic–ester bonds,^[^ ^17^, ^23^ ^]^ metal–ligand coordination,^[^ ^24^, ^25^ ^]^ and Schiff‐based imine bonds.^[^ ^26^, ^27^, ^28^ ^]^ Bao's group^[^ ^29^ ^]^ reported that when siloxane oligomers were used to construct a multiphase assembly through hydrogen bonding, the product exhibited high mechanical strength and self‐healing efficiency, but swelling remained a major concern because of the water molecules. Wang's group^[^ ^21^ ^]^ demonstrated that a new fluorinated SHP could withstand exposure to water solutions with various pH values through the external force of dipole–dipole interaction in C—F bonds. However, the self‐healing efficiency reached only 20% within 3 h; to achieve 82%, a much longer healing time—up to 1 d—was required at 50 °C. More recently, Haick's group^[^ ^30^ ^]^ presented a novel thermoplastic elastomer with water insensitivity that can be used under various harsh underwater conditions. However, the maximum stretchability of the elastomer was only 1100%, and the maximum self‐healing efficiency was only 80% after 24 h, greatly restricting their practical applications. Overall, with hydrophilic polymers, the probabilities of the reformation of dynamic bonds and successful self‐healing at cracked interfaces through H‐bonding are higher than the corresponding probabilities for hydrophobic polymers; however, hydrophilic polymers are limited by their high water sensitivity, which leads to considerable material deformation that eventually destroys the network upon swelling. By contrast, hydrophobic polymers, through their high control over van der Waals (vdW) interactions, do not swell substantially upon exposure to water, protecting them from irreversible mechanical degradation and limiting the water healing to peripheral moieties. For example, Urban's group^[^ ^31^, ^32^ ^]^ reported that a hydrophobic polymer integrated into a supramolecular network was associated with end‐to‐end distances and cohesive energy densities. Molecular dynamics simulations were performed, enabling the fine control of the vdW interaction between a water molecule and hydrophobic polymer. The investigated material thus overcomes the water‐assisted self‐healing limitation and achieves high self‐healing efficiency under ambient conditions. Despite advances in water‐based SHPs, several shortcomings remain,^[^ ^21^, ^30^, ^31^, ^32^ ^]^ including a slow self‐healing process and poor mechanical performance over repeated cutting–healing cycles. Therefore, developing an intrinsically stretchable self‐healing polymer that is waterproof, has ultrafast self‐healing efficiency, and that has superior toughness alongside long‐term underwater stability is essential.

Herein, we combined dynamic covalent imine bonds with crosslinked H‐bonds to design a highly stretchable and universally autonomous supramolecular polymeric self‐healing network. Our mechanism exploits a soft triformaldedhyde benzene (TFB) unit to conduct a weak, covalent, reversible Schiff‐base reaction involving imine metathesis to enable efficient strain energy diffusion along cracks. Furthermore, it uses a hard methylene diphenyl diisocyanate (MDI) unit to create a crosslinked network through urea‐based strong intermolecular H‐bonds to maintain elastomer robustness and elasticity stability. The polymeric network is stabilized by polydimethyl siloxane (PDMS) to achieve a balance between hydrophobicity and hydrophilicity, thereby preventing external degradation. As expected, water alters the self‐healing kinetics of the network, including energy dissipation and the chain rearrangements between hydrogen bonds and Schiff‐base imine bonds, facilitating the reversible dissociation–association between the hydrophobic cross‐links. At room temperature, our water‐assisted SHP (WASHP) recovered its initial shape with 95% healing efficiency within 1 h of sustaining mechanical damage. Moreover, it exhibited superior stretchability (9050%) and toughness (144.2 MJ m^−3^), 2.13 and 5.38 times higher than those exhibited by the pristine room‐temperature SHP (4250% and 26.8 MJ m^−3^, respectively). These favorable characteristics enable the integration of our WASHP into light‐emitting touch‐responsive devices (LETDs) capable of withstanding deformation under mechanical force (pressure, bending, and strain). Furthermore, we combined the WASHP with perovskite quantum dots (PeQDs) to fabricate a highly stable and sustainable white LED backlight device. Thus, our novel WASHP—which is eco‐friendly, economical, waterproof, and resilient—overcomes the current shortcomings and has high potential for integration into next‐generation wearable electronic devices and flexible displays.

## 2 Results and Discussion

**Figure 1:**
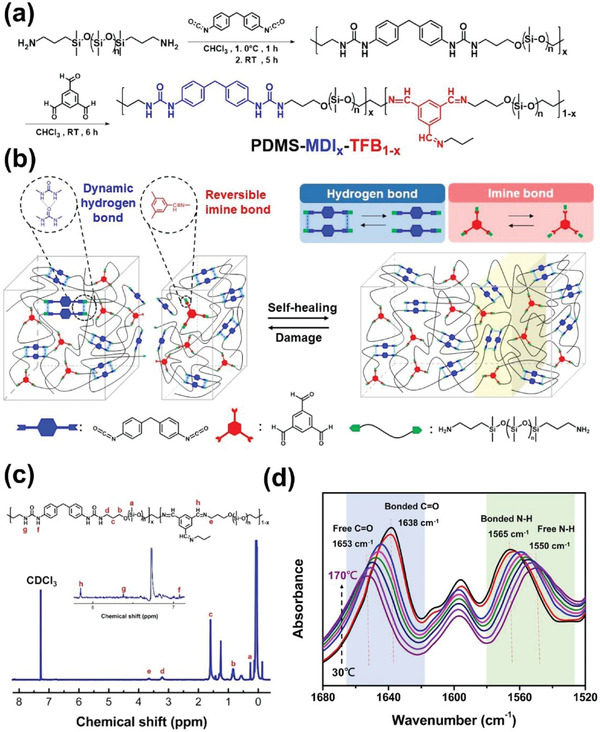
Molecular architecture of PDMS‐MDI*_x_*‐TFB_1−_ *_x_* elastomer with eco‐friendly, high toughness, stretchability, and autonomous self‐healing property. a) Synthetic route to prepare PDMS‐MDI*_x_*‐TFB_1−_ *_x_* self‐healing elastomer. b) Schematic illustration of ideal structure of PDMS‐MDI*_x_*‐TFB_1−_ *_x_* based on the synergistic effect of reversible weaker imine bonds and stronger hydrogen bonds. c) ^1^H NMR spectrum of PDMS‐MDI_0.4_‐TFB_0.6_ elastomer. d) In situ ATR‐FTIR spectra of PDMS‐MDI_0.4_‐TFB_0.6_ upon heating from 30 to 170 °C. 1660–1620 cm^−1^ (left), 1600–1520 cm^−1^ (right).

We first synthesized a series of polymers through a simple one‐pot two‐step approach involving bis(3‐aminopropyl)‐terminated PDMS (H_2_N‐PDMS‐NH_2_) and MDI. Next, the terminated NH_2_ groups of PDMS intermediate moieties were further allowed to react with the three aldehyde groups of TFB to form covalent reversible imine groups on the linear PDMS chains, forming the final product, PDMS‐MDI*_x_*‐TFB_1−_ *_x_* (**Figure** **1**a). PDMS is known to be nontoxic and biocompatible and is usually employed as a soft segment due to the loose chain packing of the polymeric backbone. Moreover, its lesser crystalline region contributes to chain flexibility and facilitates chain motion. PDMS is therefore an ideal core material for incorporation in e‐skin devices and components. MDI was selected as the hard segment due to its bulky structure and high *π*–*π* stacking density between aromatic rings, which results in strong intermolecular interaction and facilitates self‐healing in a dynamic environment. An MDI unit with a strong H‐bond can prevent notch formation and propagation within the network. The contribution of PDMS to self‐healing ability is strengthened by the TFB soft phase moieties. Reversible covalent imine bond formation occurs through the Schiff base reaction because TFB acts as a chain extender for PDMS. Moreover, zwitterionic intermediates with negatively charged imine nitrogen atoms are efficiently stabilized by the electron‐accepting aromatic ring of TFB.^[^ ^33^ ^]^ Two relevant points are presented as follows. First, TFB‐based imine bonds are stable and weak and exhibit reversible behavior in a soft environment; therefore, they can act as sacrificial bonds and enables high energy dissipation in cracked areas and ensures high reversibility. Second, transamination exchange reactions between H_2_N‐PDMS‐NH_2_ primary amines and sterically unhindered imines in organic solvents enable fast healing of cleaved imine. Therefore, stronger hydrogen bonds and reversible weaker imine bonds constitute the principle underlying the PDMS‐MDI*_x_*‐TFB_1−_ *_x_* dynamic bond network. During mechanical deformation, the synergetic effect of the hybrid network simultaneously enables favorable self‐healing and strong mechanical resistance (Figure 1b). Satisfactory self‐healing restructuring is achieved through high energy dissipation through weak reversible imine bonds dissociation, providing the structure with high toughness.

The as‐synthesized molecular structures were confirmed using nuclear magnetic resonance (^1^H NMR) and Fourier‐transform infrared spectroscopy (FTIR), with ^1^H NMR suggesting the presence of protons from both the MDI urea group and TFB imine bonds, as indicated by characteristic responses at 8.22 and 7.33 ppm, respectively. Additional peaks at 0.1 and 3.5 ppm were attributed to the methyl group in siloxane and the terminal propyl groups of the PDMS backbone, respectively (Figure 1c). PDMS‐MDI and PDMS‐TFB elastomer molecular structures were identified from their respective NMR spectra (Figures S1 and S2, Supporting Information). The FTIR analysis revealed certain structures, as indicated by vibration in the C═O (1640 cm^−1^) and C═N (1650 cm^−1^) stretching regions (Figure S3, Supporting Information), confirming the presence of the dynamic urea‐based hydrogen bonds and TFB‐based reversible covalent imine bond in PDMS‐MDI*_x_*‐TFB_1−_ *_x_*. In addition, real‐time temperature‐dependent FTIR spectra were used to monitor hydrogen bond variation in PDMS‐MDI*_x_*‐TFB_1−_ *_x_* as it was heated from 30 to 170 °C (Figure 1d) to provide insight into the behavior of the urea hydrogen bonds. The 1565 and 1550 cm^−1^ bands were ascribed to the N—H bending of hydrogen bonds and free N—H groups, indicating that the hydrogen bond transited slowly from N—H groups in the backbone to free N—H groups. The peak at 1653 cm^−1^ was assigned to the C═O stretching of free C═O groups, whereas the peak at 1638 cm^−1^ was attributed to stretching of the ordered hydrogen‐bonded C═O groups. Notably, the major region of C═O group stretching gradually decreased in intensity, disappearing at 1638 cm^−1^ and then reappearing at 1653 cm^−1^ with an increase in temperature, in accordance with results from a relevant study.^[^ ^34^ ^]^ This finding revealed that free hydrogen‐bonded C═O groups were generated from ordered C═O groups upon heating, thus demonstrating that the urea hydrogen bond balance could be steadily destroyed and reconstructed.

**Figure 2:**
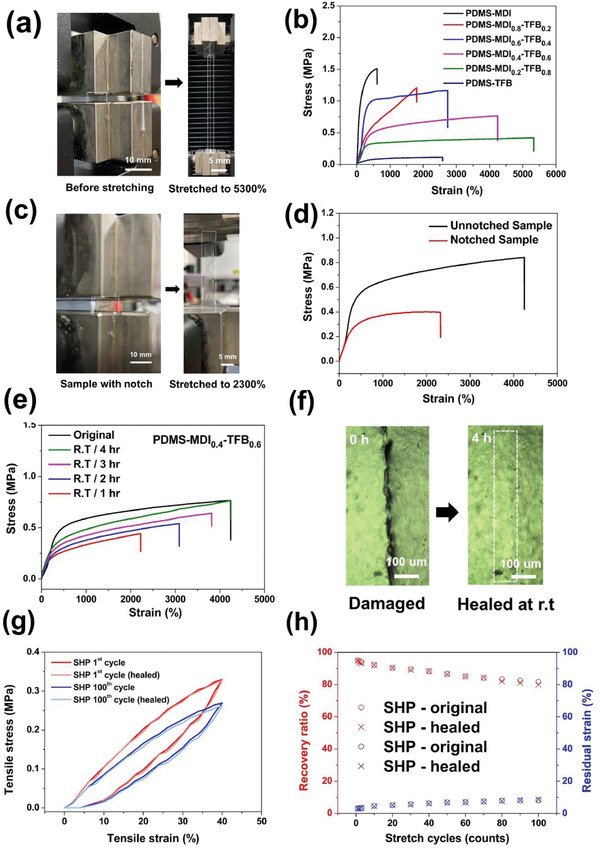
Mechanical and self‐healing properties of PDMS‐MDI*_x_*‐TFB_1−_ *_x_* elastomer film. a) Photographs of PDMS‐MDI_0.2_‐TFB_0.8_ film before stretching (left) and stretch to a strain of 5300% (right). b) Stress–strain curves of PDMS‐MDI*_x_*‐TFB_1−_ *_x_* elastomer with different ratios with a sample width of 10 mm, a thickness of 0.5 mm, and a length of 30 mm at a loading rate of 20 mm min^−1^. c) A notched‐insensitive alongside highly stretchable elastomer of PDMS‐MDI_0.4_‐TFB_0.6_ before stretching (left) and after 2300% stretching (right). The inset figure is the schematic diagram of notch location (0.5 mm in length). d) Stress–strain of the unnotched and notched PDMS‐MDI_0.4_‐TFB_0.6_ elastomer with a length of 30 mm at a loading rate of 20 mm min^−1^. e,f) Stress–strain curves of the PDMS‐MDI_0.4_‐TFB_0.6_ film healed for different time at room temperature (rt), showing that stretching ability increased is associated with the extended healing time. The optical microscope image (8 × 5 mm) (left) of PDMS‐MDI_0.4_‐TFB_0.6_ before damaged and after self‐healing 4 h at rt. g) Stress–‐strain curve of PDMS‐MDI_0.4_‐TFB_0.6_ film in cyclic stress–strain tests (40% strain). h) Recovery ratio and residual strain of PDMS‐MDI_0.4_‐TFB_0.6_ film before and after healed in cyclic stress–strain tests.

**Figure** **2** illustrates that the PDMS‐MDI*_x_*‐TFB_1−_ *_x_* film had favorable stretchability and mechanical strength. The PDMS‐MDI_0.2_‐TFB_0.8_ film was regarded as a candidate material with a reasonable ratio for a stretching test; the film could be stretched to almost 42 times its original length at a loading rate of 20 mm min^−1^ without notch formation. To determine the origin of the outstanding mechanical properties of PDMS‐MDI*_x_*‐TFB_1−_ *_x_*, PDMS‐MDI with crosslinked hydrogen bonds, and PDMS‐TFB with crosslinked imine bonds were synthesized. As shown in Figure 2b and Table S1 (Supporting Information), the Young's modulus of the PDMS‐MDI film was 0.86 MPa with a 600% strain breaking point; by contrast, the PDMS‐MDI_0.4_‐TFB_0.6_ film had a lower Young's modulus (0.18 MPa) and a higher strain breaking point (4250%) and but superior toughness (26.8 MJ m^−3^). These observations were consistent with the reduction in the Young's modulus observed upon the further insertion of TFB moieties (Table S1, Supporting Information). We assume that the mechanism underlying the synergistically enhanced mechanical strength involves simultaneous noncovalent and covalent bonding interactions, indicating that the mechanical properties of PDMS‐MDI*_x_*‐TFB_1−_ *_x_* depend on the MDI and TFB unit ratio due to the distinctive crosslinking strength of MDI‐MDI and TFB‐TFB systems. Notably, our PDMS‐MDI_0.4_‐TFB_0.6_ film could be stretched by 4250% (Figure 2b,c) and could achieve notch‐insensitive stretching (≈1–2 mm) under up to 2300% strain (Figure 2c,d); the notch was blunted without tearing when the optimal TFB/MDI unit ratio was used. This observation indicates that the concomitance of strong H‐bonds prevented notch propagation within the PDMS‐MDI film, with weak covalent imine bonds in PDMS‐TFB simultaneously breaking and dissipating the strain energy, safeguarding the structural integrity during the stretching of the cracked sample.

Furthermore, thermogravimetric analysis (Figure S4, Supporting Information) confirmed that the crosslinking density enhancement correlated with an increase in the TFB unit ratio, resulting in high supramolecular crosslinking efficiency. This demonstrates that our dynamic imine bond collection was triggered by PDMS‐TFB moieties, which caused the thermal stability threshold to expand toward the higher temperature domain. Because of its unique characteristics, our SHP could be stretched to up to 1.38 times its original length and self‐healed within only 2 h. As expected, the scar resulting from SHP tearing disappeared completely, and the polymer had higher recovered fracture stain (4200%) and high self‐healing efficiency (90%) over 4 h at room temperature (Figure 2e). These phenomena can be attributed to the synergistic interaction of multiple dynamic bonds in the supramolecular polymer network, including essential covalent imine bonds. The abundant dynamic hydrogen bonds and reversible imine bonds within the elastomer and the low *T*_g_ (≈−120 °C) of the PDMS backbone explain the favorable self‐healing efficiency. As shown in Figure S5 (Supporting Information), the SHP had the lowest *T*_g_, resulting in high mobility and facilitating the dynamic nature of the bonding healing. The findings also indicated that the *T*_g_ of the polymer was considerably lower than room temperature. One report indicated that large domain aggregations are favored by MDI with a rigid molecular structure (*π*‐aggregation) and by cooperative H‐bonds.^[^ ^29^ ^]^ Our TFB unit fulfilled two essential roles in the system, enabling energy dissipation during stretching and keeping the *T*_g_ low by preventing the excessive aggregation of MDI units. Notably, PDMS‐MDI_0.4_‐TFB_0.6_ adequately diffused away the strain energy, as indicated by mechanical hysteresis behavior in terms of both the recovery ratio and residual strain (Figure 2g,h). The area between the loading and unloading curves of the fresh SHP represents the energy dissipated per unit volume through reversible imine bond breakage. Notable hysteresis and a distinct yield point were observed in the low strain region (<40%) in the first loading–unloading cycle in the cutting–healing process; this behavior was ascribed to the reversible imine bond within the polymer network, which served as sacrificial bonds that then effectively dissipated the energy. After 100 consecutive cycles, PDMS‐MDI_0.4_‐TFB_0.6_, with a lower residual strain level (≈10%), had an excellent recovery ratio of up to 80%, and the room‐temperature‐healed SHP exhibited almost the same performance (≈2% deviation) after 100 stretching–relaxation cycles (Figure 2h). This result indicates that the mechanical reversibility is attributable to the relaxation of the polymer network through the weak reversible imine bond formation, followed by the establishment of strong hydrogen bonds. Overall, our unique PDMS‐MDI_0.4_‐TFB_0.6_ film had higher optical transmittance (83%), a short self‐healing time at room temperature (4 h) resulting in superior self‐healing efficiency (90%), and higher toughness (26.8 MJ m^−3^) than the other PDMS‐MDI*_x_*‐TFB_1−_ *_x_* polymers (Figure S6 and Table S1, Supporting Information). Its outstanding optical and mechanically reinforced properties can be leveraged in the fabrication of wearable electronic devices.

**Figure 3:**
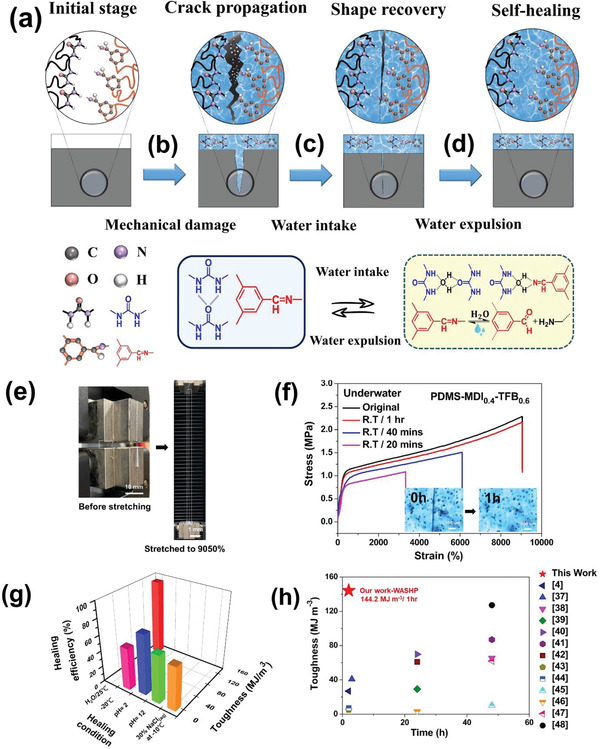
Schematic diagrams of water‐assisted self‐healing mechanism of PDMS‐MDI*_x_*‐TFB_1−_ *_x_* elastomer film. a) Illustration of reversible imine bonds and dynamic hydrogen bonds dissociation–association with water molecules upon underwater healing process. b–d) Illustration of chain motion and water‐intake following crack propagation, the chain motion within the damaged area expend, and water is getting expulsed, thereby reforming the H‐bond and covalent imine bond upon self‐healing film. e) Photographs of PDMS‐MDI_0.4_‐TFB_0.6_ film before stretching (left) and stretch to a strain of 9050% (right) after underwater healing process. f) Stress–strain curves of the PDMS‐MDI_0.4_‐TFB_0.6_ film with different underwater healing time at rt, sample width of 10 mm, a thickness of 0.5 mm, and a length of 30 mm at a loading rate of 20 mm min^−1^ (the inset displays optical microscope image (6 × 4 mm) (left) before underwater and after underwater healing within 1 h at rt). g) Self‐healing efficiency of PDMS‐MDI_0.4_‐TFB_0.6_ film in different harsh condition. h) Self‐healing time and toughness performances comparison of our work with previously self‐healing elastomers at room temperature. Water‐assisted PDMS‐MDI_0.4_‐TFB_0.6_ simultaneously exhibited the highest toughness alongside rapid self‐healing time.

Notably, our water‐assisted mechanism provides the PDMS‐MDI‐TFB elastomers with both high room‐temperature self‐healing efficiency and stretchability; they can withstand harsh conditions underwater (25 °C), in saline solution (30% NaCl solution at −10 °C), and in a strongly acidic or alkaline environment (pH = 2 or 12), as well as freezing temperatures (−20 °C). Our WASHP exhibited excellent stretchability (up to 9050%; **Figure** **3**e). Moreover, the artificial scratch had completely disappeared and the fracture stain recovered quickly to the original state over 2 h of submersion in water (25 °C), as shown in the inset of the optical microscope image presented in Figure 3f (Movie S1, Supporting Information). Compared with the pristine room‐temperature SHP, our WASHP had a considerably shorter self‐healing time (1 vs 4 h) and exhibited superior performance, by 2.13, 1.06, and 5.38 times, respectively, in tensile strain (9050% vs 4250%), self‐healing efficiency (95% vs 90%), and toughness (144.2 vs 26.8 MJ m^−3^; Figure 3e,f and Table S1, Supporting Information). Furthermore, our WASHP enables to maintain its high stretchability even at faster stretching rate up to 70 mm min^−1^ as shown in Figure S7 (Supporting Information). Moreover, our WASHP retained its self‐healing efficiency (14.1 MJ m^−3^, 53% after 8 h) even at −20 °C. This was ascribed to its viscous and fluid‐like properties and its low *T*_g_ (≤120 °C), which prevented it from freezing and promoted its self‐healing performance, due to the sufficient re‐entanglement of polymer chains at the damaged surface at even ultralow temperatures. Our WASHP also retained its self‐healing efficiency even in strongly acidic and alkaline solutions (pH 2 and 12; 20 MJ m^−3^, 75% and 16.1 MJ m^−3^, 60% after 8 h) and in 30% NaCl solution at −10 °C (16.3 MJ m^−3^, 61% after 8 h; Figure 3g and Figure S8a,b, Supporting Information). Such excellent performance indicates the reliability and stability of our WASHP in exhibiting favorable self‐healing behavior in various harsh environments without an external stimulus. This prompted us to determine the precise mechanism of underwater self‐healing. The supramolecular PDMS‐MDI‐TFB exhibited hydrophobic characteristics and a highly dense, crosslinked network. In comparison with the room‐temperature autonomous self‐healing mechanism (Figure S9, Supporting Information), we speculated that the double entropic penalty originating from PDMS conformational entropy decrease and water‐induced unfavorable vdW energetic interaction shifting would accelerate the recovery of our self‐healing polymer(Figure 3a).^[^ ^31^, ^32^, ^35^, ^36^ ^]^ First, upon mechanical damage, the polymeric chains at the damaged area undergo conformational entropic reduction caused by the polymeric segments stretching and compression within a locally reduced volume. The H‐bond array destruction provides the enthalpic penalty in the form of heat to generate the condition responsible for spontaneous self‐healing (Figure 3b). Second, upon submersion in water, the SHP sustains mechanical damage, leading to polymer chain dissociation and locally disrupted vdW interaction near the cracks due to PDMS hydrophobic nature. Localized water diffusion fast triggers an imine/amine exchange reaction and generates partial hydrolysis between the water and TFB unit due to water‐sensitive covalent imine bond. Schiff base provides a high enthalpic contribution for the reconstruction of the damaged chain at the crack area. Meanwhile, the polar–polar affinity between water and urea favors the formation of a new H‐bond array that not only competes with intramolecular urea–urea H‐bonding and vdW interactions but also forces the PDMS to undergo a conformational change (Figure 3c).^[^ ^31^, ^32^ ^]^ Therefore, the hydrophobic effect known for unfavorable entropic contribution and moderate enthalpic gain becomes a significant driving force for the accelerated recovery of the SHP to an energetically more favorable state. Third, upon the removal of the mechanical constraint, polymer chain mobility was enhanced in the damaged area, presumably due to the plasticization effect. ^[^ ^32^ ^]^ The enhanced interchain cohesive energies considerably weaken the hydrophobic polymer–water interaction, ultimately causing the expulsion of water from the system and the subsequent reestablishment of the synergistic self‐healing and reversible polymer–polymer interaction, thereby contributing to high self‐healing efficiency (Figure 3d). Overall, water molecules infiltrate and diffuse within the polymeric network at the crack area, thereby unfavorably disrupting the vdW forces and accelerating Schiff‐base hydrolysis. As a result, the enthalpic penalty offsets the entropic gain and energetically favors an accelerated self‐healing process. ^[^ ^31^, ^32^, ^35^, ^36^ ^]^ To verify our assumption, we first used a hydrogen/deuterium (H/D) exchange method to investigate the interaction between water molecules and both MDI and TFB units in the polymer network upon damage under mechanical stress. Attenuated total reflection (ATR)‐FTIR spectra were obtained (Figure S10, Supporting Information). Upon soaking in D_2_O, the peak corresponding to N—H bonding shifted from 3416 to 3428 cm^−1^, leading to the formation of N—D bonds. The peak corresponding to the C═O bond in the MDI group shifted from 1640 to 1627 cm^−1^, whereas the C═N region (1650 cm^−1^) corresponding to the TFB group slightly decreased and a new peak corresponding to free aldehyde groups appeared at 1708 cm^−1^. This indicated that D_2_O not only assisted dissociation of the H‐bond array but also triggered the imine/amine exchange reaction of imine bonds, reorganizing the original network into a new water‐driven dynamic H‐bond and imine bond crosslinking system. Our WASHP exhibited high toughness (144.2 MJ m^−3^) and a short self‐healing time (1 h at room temperature), performance superior to that reported in recent studies on stretchable and tough materials (Figure 3h).^[^ ^4^, ^29^, ^37^, ^38^, ^39^, ^40^, ^41^, ^42^, ^43^, ^44^, ^45^, ^46^, ^47^, ^48^ ^]^

**Figure 4:**
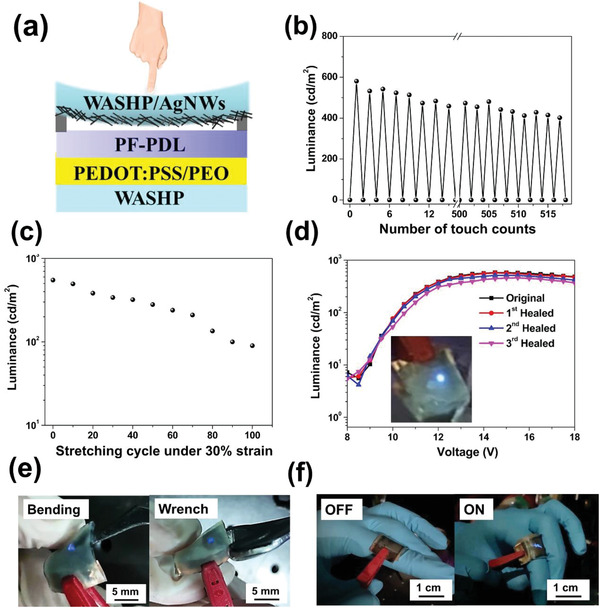
Schematic diagrams of WASHP‐based LETDs. a) Schematic illustration of LETDs structure. b) Durability test of the devices under 12 V (before and after pressure was applied onto the surface of the WASHP‐AgNW electrodes). c) Luminance–stretching cycle characteristics of the devices after repetitive stretching cycles at strains of 30%. d) Luminance characteristic of WASHP‐based LETDs during three consecutive autonomous self‐healing cycles (the inset of photograph of WASHP‐based LETDs operated at 12 V). e) Bendable and wrench LETDs (under 12 V). f) On–off light switch photograph of LETDs attached on the finger (under 12 V).

Considering this excellent performance, as a proof of concept, we exploited the quality and versatility of our SHP thin film by fabricating a WASHP‐based light‐emitting touch‐responsive device (WASHP‐LETD) with the following unique design architecture: WASHP/poly(3,4‐ethylenedioxythiophene):poly(styrenesulfonate) (PEDOT:PSS) with poly(ethylene glycol) (PEO) (PEDOT:PSS/PEO)/bio‐derived polyfluorene‐*block*‐poly(*δ*‐decanolactone) (PF‐*b*‐PDL)/WASHP with silver nanowire electrode (WASHP‐AgNWs; **Figure** **4**a). First, our previously synthesized bio‐based PF‐*b*‐PDL was selected as the emissive layer because of its favorable mechanical endurance and optoelectronic properties, including a photoluminescence quantum yield and external quantum efficiency superior to that of commercial polyfluorene (PFO).^[^ ^49^ ^]^ Second, by using our previously optimized method,^[^ ^50^ ^]^ the WASHP‐AgNW electrode was given low sheet resistance (≈22 Ω sq^−1^), high transparency (≈77%), and low surface roughness (root mean square value of 12.3 nm; Figure S11b, Supporting Information). It had satisfactory electrical performance even under bending (bending radius of 4 mm) and tensile strain (40%) over 500 cycles (Figure S12, Supporting Information). Figure 4a presents the operational mechanism of our WASHP‐LETD based on our previous design.^[^ ^49^, ^51^ ^]^ On account of the PET spacer, the additional gap between the top electrode and PF‐b‐PDL emissive layer provided high control over the touch‐responsive behavior of the device with high sensitivity. When adequate pressure was applied to the WASHP‐AgNW electrode, the resulting deformation created a contact point between the WASHP‐AgNW electrode and the emissive layer that enabled electron and hole injection under the applied voltage (Figure S13, Supporting Information).^[^ ^49^, ^51^ ^]^ As shown in Figure 4b, the device exhibited highly reproducible luminance performance after being switched on and off for 515 cycles with repeated pressure. Furthermore, the luminance–stretching cycle characteristics of the device were retained after 70 stretching cycles even after repetitive stretching at 30%, revealing the high stability of its stretchability (Figure 4c). Notably, upon three successive cutting–healing cycles (Figure 4d), the luminescence versus voltage curve retained its shape and the sheet resistance returned to its original value when the WASHP‐AgNW film had recovered its original form. This indicated that our WASHP‐LETD not only retains its luminance performance but also exhibits outstanding sustainability. The underlying principle of the AgNW percolated network behavior under cutting was confirmed using scanning electron microscopy (Figure S14, Supporting Information). First, we assumed an interplay between the ability of the SHP to control the percolation of the AgNWs upon the cutting–healing process. Second, AgNWs have high resilience to mechanical damage, enabling the torn AgNW network to repair itself through the reconnection of AgNWs over the cracks. In addition, irregular mechanical constraints are challenging for touch‐responsive devices; our device withstood mechanical deformation considerably well, with the formation of numerous contact points upon placement onto a wrench with an irregular surface, as indicated by the bright light emission finding (Figure 4e). Moreover, an on–off light switch could be realized in application to a finger joint on the basis of the bending–relaxation cycles (Figure 4f). All these results indicate that our finger‐integrated touch‐responsive LED could withstand bending to turn on a bright light because of the physical contact between the device and the joint. Overall, the WASHP‐LETD, with outstanding resilience to mechanical deformation, has high potential for use in e‐skins.

**Figure 5:**
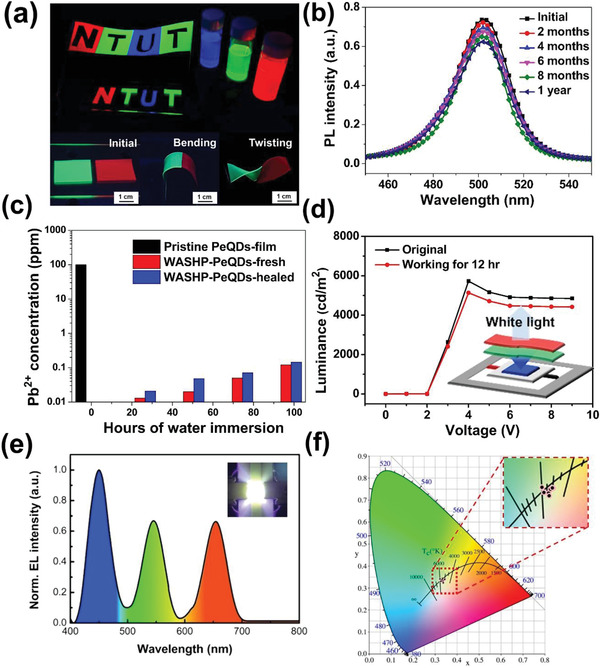
Schematic diagrams of WASHP‐based PeQDs WLED and backlight. a) Optical images of PeQDs solution (CsPbX_3_ (X = Cl, Br, I)) and WASHP‐based PeQDs film under a 365 nm UV lamp, followed by self‐healing and mechanical test including bending and twisting. b) PL spectra of WASHP‐based PeQDs film immersed underwater with different healing time. c) Variation of Pb leakage concentrations over time of our WASHP‐based PeQDs film in the contaminated water measured by ICP‐MS spectra. d) Luminance emission of bilayer WASHP‐based PeQDs WLED (the inset picture displays the configuration of the prototype WLED device). e) EL spectrum of the WASHP‐based PeQDs WLED (3 V, 6 mA), represented in the inset picture. f) CIE color coordinates demonstrated pure white color rendering of our devices.

The inorganic cesium perovskite crystal structure has recently emerged as a promising optoelectronic material due to its high color retention, high luminous efficiency, and favorable emission quality properties and has excellent performance in LED electronics.^[^ ^52^, ^53^, ^54^, ^55^, ^56^ ^]^ However, its application is limited by a major bottleneck concerning its optical stability and sustainability against various environmental factors involving air, humidity, water, and organic solvents.^[^ ^52^, ^57^ ^]^ To address those issues, surface engineering, nanofiber blending, and polymer encapsulation approaches have been explored to improve the stability and optical performance of the perovskite.^[^ ^52^, ^57^, ^58^ ^]^ Inspired by advanced encapsulation approaches, we present an original polymeric encapsulation approach in which our SHP encapsulates cesium lead halide perovskite quantum dots CsPbX_3_ (X = Cl, Br, or I), denoted PeQDs. Herein, we synthesized PeQDs using our previous hot injection method prior to embedding them in the WASHP composite. The UV absorption and photoluminescence (PL) of CsPbX_3_ (X = Cl, Br, or I) was tuned by partially replacing the Br^−^ anion with Cl^−^ or I^−^ (Figure S15, Supporting Information). The X‐ray diffraction patterns of the anion hybrid PeQDs, including CsPb(Cl_0.5_Br_0.5_)_3_ QDs, CsPbBr_3_ QDs, and CsPb(Br_0.8_I _0.2_)_3_ QDs, coincide favorably with those of the cubic CsPbBr_3_ phase (JCPDS No. 75‐0412; Figure S16, Supporting Information). Our WASHP‐QD composite was found to have high long‐term stability (Figure 5b; Figures S17 and S18, Supporting Information). The tolerance of the WASHP‐QD composite to boiling water was investigated by obtaining the PL spectra, and the PL of the pristine CsPbBr_3_ QDs was quenched within 2 min (Figure S19, Supporting Information), whereas that of the WASHP‐QD composite decreased by only 16% from its initial emissivity. This indicates high long‐term stability without substantial degradation, even in a boiling water environment over a one‐year period (**Figure** **5**b). These outstanding features—long‐term stability and reliability—represent a considerable improvement in perovskite stability over that reported in recent studies (Table S2, Supporting Information). We further investigated the Pb^2+^ toxicity of pristine CsPbBr_3_ QDs and the WASHP‐QD composite before and after healing at room temperature (Figure 5c). The WASHP‐QD composite exhibited outstanding long‐term stability and limited Pb leakage (0.1 ppm) even after 3 d of immersion in boiling water, superior to that of the pristine CsPbBr_3_ QDs film. Moreover, the Pb leakage rate of WASHP‐fresh (2.65 × 10^−4^ cm^2^) and WASHP‐healed (3.26 × 10^−4^ cm^2^) both exhibited much lower than pristine CsPbBr_3_ QDs film (21.8 cm^2^) as shown in Table S3 (Supporting Information). This indicates that the composite strongly suppressed the Pb leakage, and that and room temperature healing was facilitated by the reduced diffusion of oxygen and moisture in the polymeric matrix. Notably, our WASHP‐QD composite, emitting different colors under irradiation with a UV lamp (375 nm), retained not only its toughness and self‐healing behavior but also its bright luminescence under bending and twisting in a mechanical test (Figure 5a). Its luminescence performance was the same even after the composite was dipped in boiling water in a stretching test (Movie S2, Supporting Information). The hydrogen and imine bonds involved in the elasticity and energy dissipation mechanism did not interact with perovskite nor were they sterically impeded (Figure S20, Supporting Information), meaning that these selective interactions resulted in a material that properly encapsulated the perovskite quantum dots without disrupting the self‐healing mechanism, as presented in photographs of the self‐healing process (Figure S21, Supporting Information). For demonstration of the novel concept, a unique prototype of a WASHP‐based PeQD white LED device was assembled through the combination of a commercial InGaN blue LED chip (*λ*_max_ = 450 nm) and the WASHP‐QD composite (green and red in Figure 5d). The initial WLED device had a low operating voltage of 3 V with a maximum luminance of 5730 cd m^−2^ at 4 V; it retained its performance under a continuous operating voltage of 3 V and current of 6 mA over 12 h (Figure 5d). Our WASHP‐QD composite ensured the long‐term operating stability of the device with regard to the effective radiative recombination (*τ*_av_ = 5.05) and exciton binding energy (≈85.91 meV), the values of which substantially surpassed those of the pristine PeQDs (4.07 and ≈51.23 meV, respectively), corresponding to temperature‐dependent PL (Figure S22, Supporting Information) and time‐resolved PL (Figure S23 and Table S4, Supporting Information).^[^ ^52^, ^59^ ^]^ Furthermore, white light electroluminescence spectra and high‐quality color rendering with CIE color coordinates (0.33, 0.33) highlights the high efficacy of our WASHP‐QD composite (Figure 5e,f). Our WASHP‐QD composite has high potential for use in backlight and color‐converting films for next‐generation display devices.

## 3 Conclusion

In sum, we presented a novel universal room‐temperature autonomous self‐healing mechanism based on highly synergistically covalent weak Schiff‐base imine bonds with strong cross‐linked H‐bonds. The water‐assisted reversible dissociation–association of noncovalent bonds provides our PDMS‐MPI‐TFB film with excellent self‐healing properties, including rapid self‐healing kinetic behavior, as well as high stretchability. Moreover, upon damage under mechanical stress, the pristine room‐temperature SHP has a short self‐healing time (4 h) and high stretchability (4250%), self‐healing efficiency (90%), and toughness (26.8 MJ m^−3^). Notably, our WASHP had a self‐healing time of only 1 h as well as excellent overall performance, including a tensile strain of 9050%, self‐healing efficiency of 95%, and toughness of 144.2 MJ m^−3^. On the basis of this promising material, we developed an innovative approach for integrating a WASHP‐LETD and WASHP‐QD white LED backlight application for use in wearable electronics. The WASHP‐LETD can adhere to any rough surface and has controlled luminance emission owing to its outstanding ability to withstand mechanical deformation. Our WASHP‐QD composite effectively suppresses Pb leakage (0.1 ppm), resulting in high long‐term stability, including in boiling water over a one‐year period. As a proof of concept, a unique prototype of a WASHP‐QD white LED backlight was fabricated through the integration of a blue LED chip and the WASHP‐QD composite. Our multifunctional WASHP has extensive potential applications in wearable electronic devices and flexible displays.

## 4 Experimental Section

Full experimental details are provided in the Supporting Information.

## Conflict of Interest

The authors declare no conflict of interest.

## Supporting information

Supporting Information

Supplemental Movie 1

Supplemental Movie 2

## Data Availability

The data that support the findings of this study are available from the corresponding author upon reasonable request.

## References

[1] A. Chortos , J. Liu , Z. Bao , Nat. Mater. 2016, 15, 937. 27376685 10.1038/nmat4671

[2] Y. Liu , K. He , G. Chen , W. R. Leow , X. Chen , Chem. Rev. 2017, 117, 12893. 28991450 10.1021/acs.chemrev.7b00291

[3] S. Wang , J. Y. Oh , J. Xu , H. Tran , Z. Bao , Acc. Chem. Res. 2018, 51, 1033. 29693379 10.1021/acs.accounts.8b00015

[4] S. M. Kim , H. Jeon , S. H. Shin , S. A. Park , J. Jegal , S. Y. Hwang , D. X. Oh , J. Park , Adv. Mater. 2018, 30, 1705145. 10.1002/adma.201705145 29131415

[5] B. C. Tee , C. Wang , R. Allen , Z. Bao , Nat. Nanotechnol. 2012, 7, 825. 23142944 10.1038/nnano.2012.192

[6] J. Sun , X. Pu , M. Liu , A. Yu , C. Du , J. Zhai , W. Hu , Z. L. Wang , ACS Nano 2018, 12, 6147. 29851468 10.1021/acsnano.8b02479

[7] X. Liu , G. Su , Q. Guo , C. Lu , T. Zhou , C. Zhou , X. Zhang , Adv. Funct. Mater. 2018, 28, 1706658.

[8] P. Wu , H. Cheng , X. Wang , R. Shi , C. Zhang , M. Arai , F. Zhao , Green Chem. 2021, 23, 552.

[9] Y.‐H. Li , W.‐J. Guo , W.‐J. Li , X. Liu , H. Zhu , J.‐P. Zhang , X.‐J. Liu , L.‐H. Wei , A.‐L. Sun , Chem. Eng. J. 2020, 393, 124583.

[10] X. Zhang , S. Wang , Z. Jiang , Y. Li , X. Jing , J. Am. Chem. Soc. 2020, 142, 21852. 33332118 10.1021/jacs.0c10244

[11] X. Yang , G. Liu , L. Peng , J. Guo , L. Tao , J. Yuan , C. Chang , Y. Wei , L. Zhang , Adv. Funct. Mater. 2017, 27, 1703134.

[12] M. Burnworth , L. Tang , J. R. Kumpfer , A. J. Duncan , F. L. Beyer , G. L. Fiore , S. J. Rowan , C. Weder , Nature 2011, 472, 334. 21512571 10.1038/nature09963

[13] W. Fan , Y. Jin , L. Shi , W. Du , R. Zhou , S. Lai , Y. Shen , Y. Li , ACS Appl. Mater. Interfaces 2020, 12, 6383. 31903744 10.1021/acsami.9b18985

[14] P. Song , H. Qin , H. L. Gao , H. P. Cong , S. H. Yu , Nat. Commun. 2018, 9, 2786. 30018323 10.1038/s41467-018-05238-w PMC6050250

[15] R. Du , Z. Xu , C. Zhu , Y. Jiang , H. Yan , H. C. Wu , O. Vardoulis , Y. Cai , X. Zhu , Z. Bao , Q. Zhang , X. Jia , Adv. Funct. Mater. 2019, 30, 1907139.

[16] Y. Yanagisaw , Y. Nan , K. Okuro , T. Aida , Science 2018, 359, 72. 29242235 10.1126/science.aam7588

[17] B. K. Ahn , D. W. Lee , J. N. Israelachvili , J. H. Waite , Nat. Mater. 2014, 13, 867. 25064231 10.1038/nmat4037

[18] Y. Yang , M. W. Urban , Angew. Chem. 2014, 53, 12142. 25220903 10.1002/anie.201407978

[19] M. Liu , P. Liu , G. Lu , Z. Xu , X. Yao , Angew. Chem. 2018, 57, 11242. 29993173 10.1002/anie.201805206

[20] B. Willocq , F. Khelifa , J. Odent , V. Lemaur , Y. Yang , P. Leclere , J. Cornil , P. Dubois , M. W. Urban , J. M. Raquez , ACS Appl. Mater. Interfaces 2019, 11, 46176. 31736297 10.1021/acsami.9b16858

[21] Y. Cao , H. Wu , S. I. Allec , B. M. Wong , D. S. Nguyen , C. Wang , Adv. Mater. 2018, 30, 1804602. 10.1002/adma.201804602 30368928

[22] J. Sedo , J. Saiz‐Poseu , F. Busque , D. Ruiz‐Molina , Adv. Mater. 2013, 25, 653. 23180685 10.1002/adma.201202343

[23] Z. Shafiq , J. Cui , L. Pastor‐Perez , V. San Miguel , R. A. Gropeanu , C. Serrano , A. del Campo , Angew. Chem. 2012, 51, 4332. 22461306 10.1002/anie.201108629

[24] N. N. Xia , X. M. Xiong , J. Wang , M. Z. Rong , M. Q. Zhang , Chem. Sci. 2016, 7, 2736. 28660049 10.1039/c5sc03483c PMC5477145

[25] J. Li , H. Ejima , N. Yoshie , ACS Appl. Mater. Interfaces 2016, 8, 19047. 27377859 10.1021/acsami.6b04075

[26] P. Taynton , K. Yu , R. K. Shoemaker , Y. Jin , H. J. Qi , W. Zhang , Adv. Mater. 2014, 26, 3938. 24677455 10.1002/adma.201400317

[27] S. Delpierre , B. Willocq , G. Manini , V. Lemaur , J. Goole , P. Gerbaux , J. Cornil , P. Dubois , J.‐M. Raquez , Chem. Mater. 2019, 31, 3736.

[28] T. W. Lin , S. H. Hsu , Adv. Sci. 2020, 7, 1901388. 10.1002/advs.201901388 PMC7001655 32042553

[29] J. Kang , D. Son , G. N. Wang , Y. Liu , J. Lopez , Y. Kim , J. Y. Oh , T. Katsumata , J. Mun , Y. Lee , L. Jin , J. B. Tok , Z. Bao , Adv. Mater. 2018, 30, 1706846. 10.1002/adma.201706846 29424026

[30] M. Khatib , O. Zohar , W. Saliba , S. Srebnik , H. Haick , Adv. Funct. Mater. 2020, 30, 1910196.

[31] M. W. Urban , D. Davydovich , Y. Yang , T. Demir , Y. Zhang , L. Casabianca , Science 2018, 362, 220. 30309952 10.1126/science.aat2975

[32] D. Davydovich , M. W. Urban , Nat. Commun. 2020, 11, 5743. 33184268 10.1038/s41467-020-19405-5 PMC7665198

[33] P. Kovaricek , J. M. Lehn , J. Am. Chem. Soc. 2012, 134, 9446. 22582711 10.1021/ja302793c

[34] Y. Song , Y. Liu , T. Qi , G. L. Li , Angew. Chem. 2018, 57, 13838. 30144244 10.1002/anie.201807622

[35] Y. Yang , M. W. Urban , Chem. Soc. Rev. 2013, 42, 7446. 23864042 10.1039/c3cs60109a

[36] C. C. Hornat , M. W. Urban , Prog. Polym. Sci. 2020, 102, 101208.

[37] J. Xu , W. Chen , C. Wang , M. Zheng , C. Ding , W. Jiang , L. Tan , J. Fu , Chem. Mater. 2018, 30, 6026.

[38] D. Wang , J. Xu , J. Chen , P. Hu , Y. Wang , W. Jiang , J. Fu , Adv. Funct. Mater. 2019, 30, 1907109.

[39] J. C. Lai , X. Y. Jia , D. P. Wang , Y. B. Deng , P. Zheng , C. H. Li , J. L. Zuo , Z. Bao , Nat Commun. 2019, 10, 1164. 30858371 10.1038/s41467-019-09130-z PMC6411951

[40] Y. Miwa , J. Kurachi , Y. Kohbara , S. Kutsumizu , Commun. Chem. 2018, 1, 5.

[41] L. Zhang , Z. Liu , X. Wu , Q. Guan , S. Chen , L. Sun , Y. Guo , S. Wang , J. Song , E. M. Jeffries , C. He , F. L. Qing , X. Bao , Z. You , Adv. Mater. 2019, 31, 1901402. 10.1002/adma.201901402 30977571

[42] Y. Wang , X. Liu , S. Li , T. Li , Y. Song , Z. Li , W. Zhang , J. Sun , ACS Appl. Mater. Interfaces 2017, 9, 29120. 28795571 10.1021/acsami.7b08636

[43] J. Xu , P. Chen , J. Wu , P. Hu , Y. Fu , W. Jiang , J. Fu , Chem. Mater. 2019, 31, 7951.

[44] P.‐F. Cao , B. Li , T. Hong , J. Townsend , Z. Qiang , K. Xing , K. D. Vogiatzis , Y. Wang , J. W. Mays , A. P. Sokolov , T. Saito , Adv. Funct. Mater. 2018, 28, 1800741.

[45] Q. Zhang , S. Niu , L. Wang , J. Lopez , S. Chen , Y. Cai , R. Du , Y. Liu , J. C. Lai , L. Liu , C. H. Li , X. Yan , C. Liu , J. B. Tok , X. Jia , Z. Bao , Adv. Mater. 2018, 30, 1801435. 10.1002/adma.201801435 29978512

[46] W. Yao , X. Chen , Q. Tian , C. Luo , X. Zhang , H. Peng , W. Wu , Chem. Eng. J. 2020, 384, 123375.

[47] J. H. Xu , S. Ye , C. D. Ding , L. H. Tan , J. J. Fu , J. Mater. Chem. A 2018, 6, 5887.

[48] Z. Shi , J. Kang , L. Zhang , ACS Appl. Mater. Interfaces 2020, 12, 23484. 32343136 10.1021/acsami.0c04414

[49] D.‐H. Jiang , B. J. Ree , T. Isono , X.‐C. Xia , L.‐C. Hsu , S. Kobayashi , K. H. Ngoi , W.‐C. Chen , C.‐C. Jao , L. Veeramuthu , T. Satoh , S. H. Tung , C.‐C. Kuo , Chem. Eng. J. 2021, 418, 129421.

[50] F. C. Liang , Y. W. Chang , C. C. Kuo , C. J. Cho , D. H. Jiang , F. C. Jhuang , S. P. Rwei , R. Borsali , Nanoscale 2019, 11, 1520. 30620020 10.1039/c8nr08819e

[51] D. H. Jiang , Y. C. Liao , C. J. Cho , L. Veeramuthu , F. C. Liang , T. C. Wang , C. C. Chueh , T. Satoh , S. H. Tung , C. C. Kuo , ACS Appl. Mater. Interfaces 2020, 12, 14408. 32118411 10.1021/acsami.9b23291

[52] L. N. Quan , B. P. Rand , R. H. Friend , S. G. Mhaisalkar , T. W. Lee , E. H. Sargent , Chem. Rev. 2019, 119, 7444. 31021609 10.1021/acs.chemrev.9b00107

[53] J. Song , J. Li , L. Xu , J. Li , F. Zhang , B. Han , Q. Shan , H. Zeng , Adv. Mater. 2018, 30, 1800764. 10.1002/adma.201800764 29888521

[54] D. Chen , X. Chen , Z. Wan , G. Fang , ACS Appl. Mater. Interfaces 2017, 9, 20671. 28569064 10.1021/acsami.7b05429

[55] W.‐C. Chen , Y.‐H. Fang , L.‐G. Chen , F.‐C. Liang , Z.‐L. Yan , H. Ebe , Y. Takahashi , T. Chiba , J. Kido , C.‐C. Kuo , Chem. Eng. J. 2021, 414, 128866.

[56] Z.‐L. Yan , J.‐S. Benas , C.‐C. Chueh , W.‐C. Chen , F.‐C. Liang , Z.‐X. Zhang , B.‐H. Lin , C.‐J. Su , T. Chiba , J. Kido , C.‐C. Kuo , Chem. Eng. J. 2021, 414, 128774.

[57] Y. Wei , Z. Cheng , J. Lin , Chem. Soc. Rev. 2019, 48, 310. 30465675 10.1039/c8cs00740c

[58] C. C. Lin , D. H. Jiang , C. C. Kuo , C. J. Cho , Y. H. Tsai , T. Satoh , C. Su , ACS Appl. Mater. Interfaces 2018, 10, 2210. 29308867 10.1021/acsami.7b15989

[59] M. Lu , Y. Zhang , S. Wang , J. Guo , W. W. Yu , A. L. Rogach , Adv. Funct. Mater. 2019, 29, 1902008.

