# Supplementary material for: Super Tough and Spontaneous Water‐Assisted Autonomous Self‐Healing Elastomer for Underwater Wearable Electronics
Source: Adv Sci (Weinh). 2021 Sep 14;8(21):2102275. doi: 10.1002/advs.202102275 (PMC8564429; doi:10.1002/advs.202102275)
Supplement: Supplementary file 1 — Supporting Information [file ADVS-8-2102275-s002.pdf]

© 2021 Wiley-VCH GmbH

ADVANCED SCIENCE
Open Access

Supporting Information

for *Adv. Sci.*, DOI: 10.1002/advs.202102275

Super Tough and Spontaneous Water-Assisted Autonomous Self-Healing Elastomer for Underwater Wearable Electronics

Cyuan-Lun He[a,+], Fang-Cheng Liang[a,+,*], Loganathan Veeramuthu[a], Chia-Jung Cho[a], Jean-Sebastien Benas[a], Yung-Ru Tzeng[a], Yen-Lin Tseng[a], Wei-Cheng Chen[a], Alina Rwei[b], Chi-Ching Kuo[a,*]

Supporting Information

Super Tough and Spontaneous Water-Assisted Autonomous Self-Healing Elastomer for Underwater Wearable Electronics

Cyuan-Lun He[a,+], Fang-Cheng Liang[a,+,*], Loganathan Veeramuthu[a], Chia-Jung Cho[a], Jean-Sebastien Benas[a], Yung-Ru Tzeng[a], Yen-Lin Tseng[a], Wei-Cheng Chen[a], Alina Rwei[b], Chi-Ching Kuo[a,*]

[a] Institute of Organic and Polymeric Materials, Research and Development Center of Smart Textile Technology, National Taipei University of Technology, No. 1, Sec. 3, Chung-Hsiao East Road., Taipei, 10608, Taiwan

[b] Department of Chemical Engineering, Delft University of Technology, 2629 HZ Delft, Netherlands

+C.-L. He and F.-C. Liang contributed equally to this work.

*Author to whom all correspondence should be addressed

Tel.: 886-2-27712171*2407; Fax: 886-2-27317174

Correspondence to: Prof. C.-C. Kuo (E-mail: kuocc@mail.ntut.edu.tw)

Dr. F.-C. Liang (E-mail: frank62112003@mail.ntut.edu.tw)

Synthesis of Perovskite QDs:

Cs_2CO_3 (203.5 mg) and oleic acid (625µL) made completely soluble in 1-octadecene (10 mL) in 25-mL two-neck round bottom flask. The solution under vacuum was heated to 120 °C for 1 h under stirring. The reaction flask was N_2 filled, and the temperature was further increased to 150 °C. Another two neck flask was loaded with $PbBr_2$ (138.18 mg) and 1-octadecene (10 mL) under vacuum at 120 °C for 1 h under stirring followed by N_2 gas loading. Oleylamine (1 mL) and oleic acid (1 mL) were added to stabilize the lead precursors and it is subjected to 170 °C, followed by rapid injection of 0.8 mL of Cs-oleate solution. The reaction is quenched in an ice bath immediately after 5 s to form the green $CsPbBr_3$ QD solution. Then, colloidal QD solution was cooled and centrifuged at 4000 rpm for 10 min to separate the supernatant and precipitates. Pure QD solution was obtained by dispersing the precipitate with toluene. For red QD preparation, PbI_2 and $PbBr_2$ (8:2) mixture was utilized instead of $PbBr_2$ precursor and the entire synthetic procedure remains the same.

Self-healable conductive WASHP/AgNW electrode fabrication:

SHP (1.0 g) was dissolved in chloroform (10.0 ml) and poured on top of the PTFE substrate and consequently cured at room temperature to achieve 3cm x 6cm with thickness ~0.5 mm. The SHP substrates was further immersing into water for 10 mins

and then directly plasma treated (60 s). Based on our previous method,[47] the AgNWs at concentrations of 0.066% were prepared and dispersed in isopropanol then further put to the container. The air brush of a spray gun (FUSO SEIKI, 033G-Double Action) was set at 45 psi with a spraying distance of 10 cm from the surface of the WASHP substrate within 30s and annealed at 40 °C for 30 mins. Subsequent optimization process led to the formation of conductive percolative AgNWs adhered to the drop-cast SHP film, which was peeled from the PTFE substrate to utilize as transparent composite electrodes.

Fabrication of stretchable touch responsive WASHP LED device:

SHP substrate were prepared by drop-coating onto the PTFE substrate and being cured at room temperature to achieve 3 cm x 6 cm with thickness ~0.5 mm. The SHP substrates were further immersing into water for 10 mins and then treated with oxygen plasma for 2 mins. Based on our previous method,[48] The PEDOT:PSS / PEO solution was spin-coated onto the treated WASHP substrate at 1000 rpm for 30 s. Subsequently, the film was annealed at 80 °C for 15 facilitated the thin film formation. After cooling, spin-coated with the PF-b-PDL layer at 1000 rpm for 30 s and annealed at 70 °C for 10 min. The spacer (PET) was introduced onto the emissive layer. The prepared SHP/AgNW electrode was then faced down and stacked onto the emissive layer that plays the role of a cathode.

Preparation of WASHP-QDs White LED device:

SHP (1.0 g) was dissolved in toluene (10.0ml) and blended into green/red perovskites QDs (10 mg/ml) 1.0 ml solution to form SHP QD solution. The SHP QD solution was stirred at 30 °C for 3 hours, and poured into poly (tetrafluoroethylene) (PTFE) substrate and consequently cured at 60 °C. After the SHP-QDs composite immersing into water for 10 mins in order to elevate the mechanical property, the white LEDs (WLEDs) were fabricated by stacking green and red WASHP QDs composites on top of the commercial blue LED chips (450 nm).

Characterizations:

Proton nuclear magnetic resonance spectroscopy (^{1}H NMR) was recorded on a Bruker Fourier 300 (300 MHz) spectrometer (Bruker, Germany) with chloroform-d as solvent and TMS as internal standard. Fourier transform infrared spectroscopy (FT-IR) was performed on a spectrum Two (PerkinElmer, UK) in the region of 4000–400 cm^{-1} with 16 scans at a resolution of 4 cm^{-1}. Gel permeation chromatography (GPC) experiments were performed on a RI-2031,plus (JASCO, Japan) solvent/sample module with an optimal refractive index detector and THF was used as an elution solvent at a flow rate of 1.0 mL min^{-1}. Thermogravimetric analysis (TGA) instrument was performed on a TG-209 (NETZSCH, U.S.) was used to measure the thermal decomposition stability. The XRD patterns of the prepared films were characterized by PANalytical diffractometer

(X' Pert3 Powder). Dynamic mechanical analysis (DMA) was performed on an EXSTAR 6000 (Techmax Technical Co., Ltd., U.S.) was used to measure viscoelastic properties. A tensile test QC 508 (Cometech Testing Machines Co., Ltd., Taiwan) was conducted for determining the mechanical properties. The measurements were conducted at different loading rate (20 mm min^{-1}, 50 to 70 mm min^{-1}). The surface roughness was measured by atomic force microscopy (AFM, Park Systems, XE-100TM, USA). Field emission scanning electron microscope (FE-SEM) was performed on a S4800 (HITACHI, Japan) observed before and after healing. The sheet resistance of the WASHP-AgNW electrodes was measured using a four-point probe technique and a Keithley 2400 source meter. An Ultraviolet–visible spectrophotometer was performed on a V-730 (JASCO, Japan) was utilized to test the light transmittance of the elastomer sample. The Photoluminescence spectra were measured by a Fluoromax spectrofluorometer (Horiba, France), and the polymer films were excited at wavelengths of 365 nm. Photoluminescence quantum yield (PLQY) were obtained using a Research Grade Spectrofluorometer system (FP-8500, JASCO). Time-resolved lifetime (TR-PL, Hamamatsu C11367 Quantaurus-Tau), Temperature-dependence photoluminescence (Temp-PL) was obtained by using a pulsed diode-laser (PicoQuant LDH-D-C-375) and the excitation wavelength was 375 nm. The Commission Internationale de l'éclairage (CIE) color coordinates of the LED spectra were measured by PR670 spectroradiometer (Titan Electro-Optics Co., Ltd., Taiwan).

Toughness and Self-healing efficiency calculation:

The toughness (E) was calculated by integration of the area under the stress-strain curves.

The self-healing efficiency was calculated as follows:

$$\mathrm{Self-healing\ efficiency\ \%} = \frac{\mathrm{E}_{healed}}{\mathrm{E}_{fresh}}"\boldsymbol{x100}$$

Where E-fresh and E-healed corresponds to the tensile energy (E) for fresh SHP material and healed SHP material.

Recovery ratio and residual strain % calculation:

The Energy dissipation can be measured from the area under the cyclic tensile stress strain curve. The energy dissipation of the first cycle (E_0) and the final cycle (E_f) can be measured and it is applied in the following equation to derive the recovery ratio %.

$$\mathrm{Recovery\ ratio\ \%} = \frac{\mathrm{E}_f}{\mathrm{E}_0}\mathbf{x100}$$

The residual strain % is defined as the ratio of the difference in initial length (ΔL) to the initial length (L_0) of the material after straining it to specified extent for several cycles.

$$\mathrm{Residual\ strain\ ratio\ \%} = \frac{\Delta L}{L_0}\mathbf{x100}$$

Time resolved photoluminescence Average lifetime calculation:

Time resolved PL curves were fitted by biexponential decay function as follows,

$$F(t) = \sum_{i=1}^{n} A_i e^{-\frac{t}{\tau_i}} \qquad (n=2) \qquad (1)$$

$$\tau_{avg} = \frac{\sum_{i=1}^{n} A_i \tau_i^2}{\sum_{i=1}^{n} A_i \tau_i} \qquad (n=2) \qquad (2)$$

where *F*(*t*) is the time-dependent PL intensity; A_1, and A_2 are the intensities at *t* = 0; and τ_1 ,and τ_2 are the non-radiative and radiative components of PL decay lifetime. After solving the equation 1, we utilized A1.A2, τ_1 and τ_2 to deduce the average lifetime using equation 2.

Temperature dependent photoluminescence exciton binding energy calculation:

Integrated PL intensities were recorded with different temperatures represents the temperature dependent PL curves. By fitting with Arrhenius equation (4), we obtain the exciton binding energy from temperature-dependent PL intensity; where I_0 is the intensity at lowest temperature (77 K), E_b is the exciton binding energy, and k is the Boltzmann constant.

$$I(T) = \frac{I_0}{1 + e^{-\frac{E_b}{kT}}} \qquad (4)$$

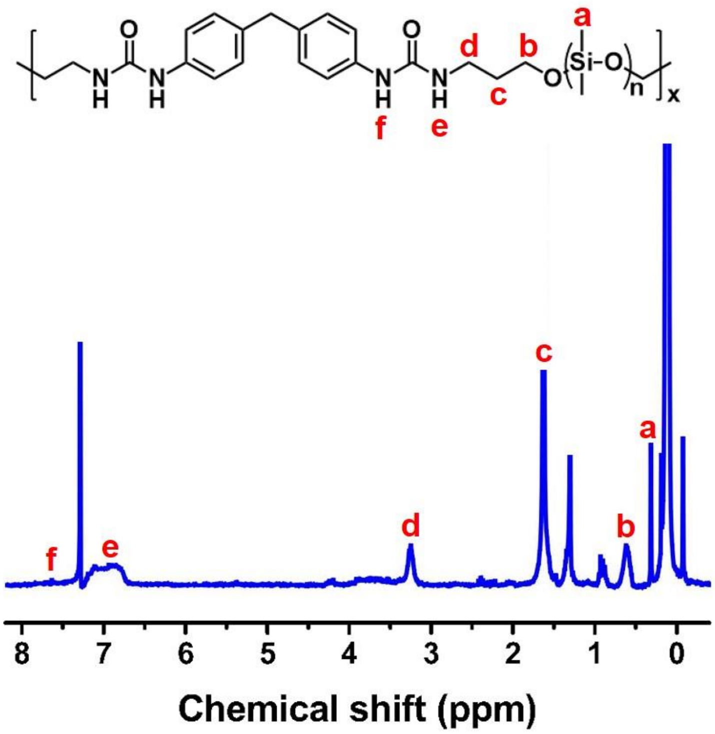

Figure S1. ^{1}H-NMR spectrum of PDMS-MDI.

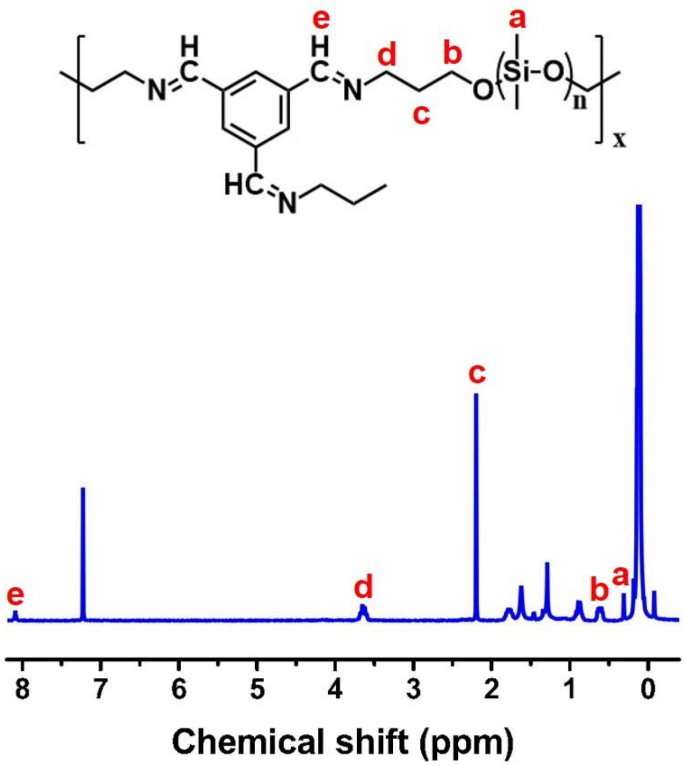

Figure S2. ^{1}H-NMR spectrum of PDMS-TFB.

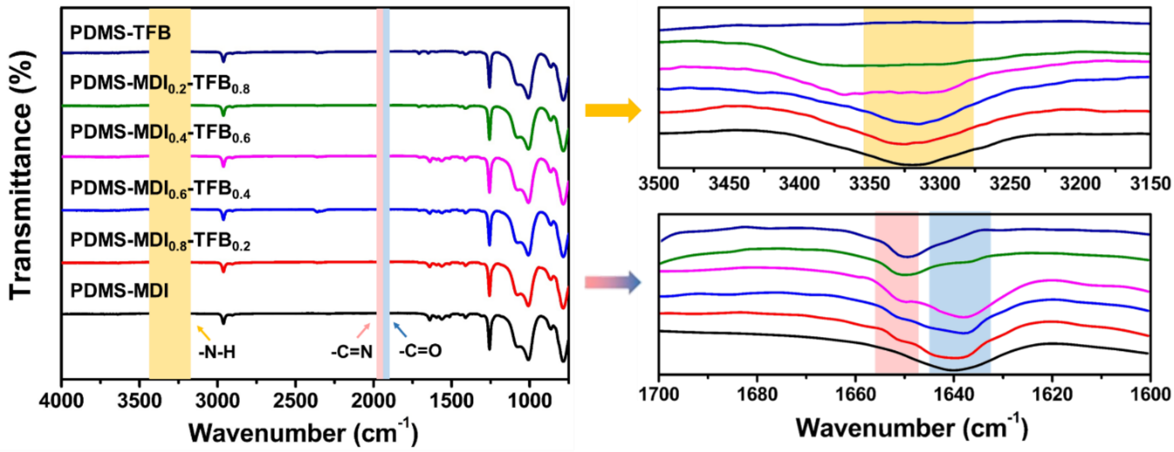

Figure S3. FT-IR spectra of different ratio PDMS-MDI_x-TFB_{1-x} elastomers.

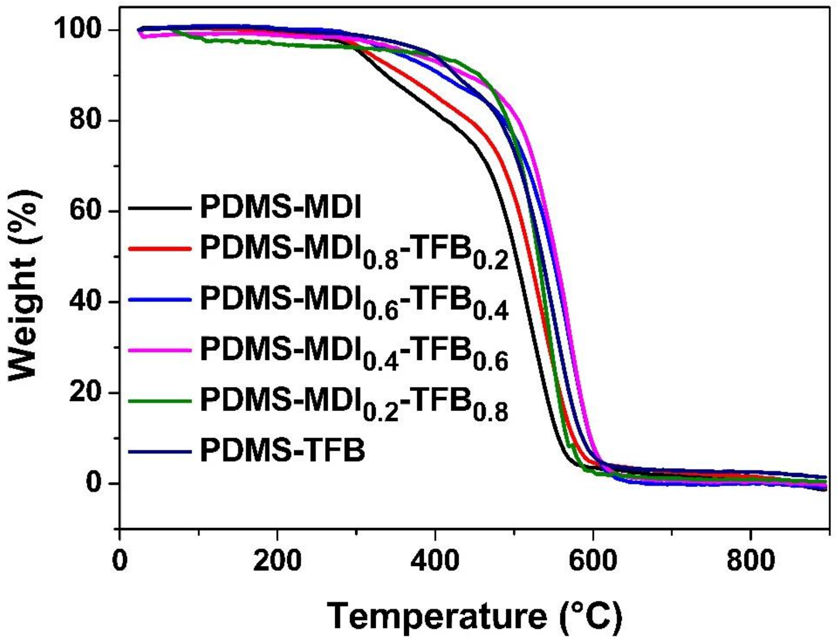

Figure S4. TGA curves of PDMS-MDI_x-TFB_{1-x}.

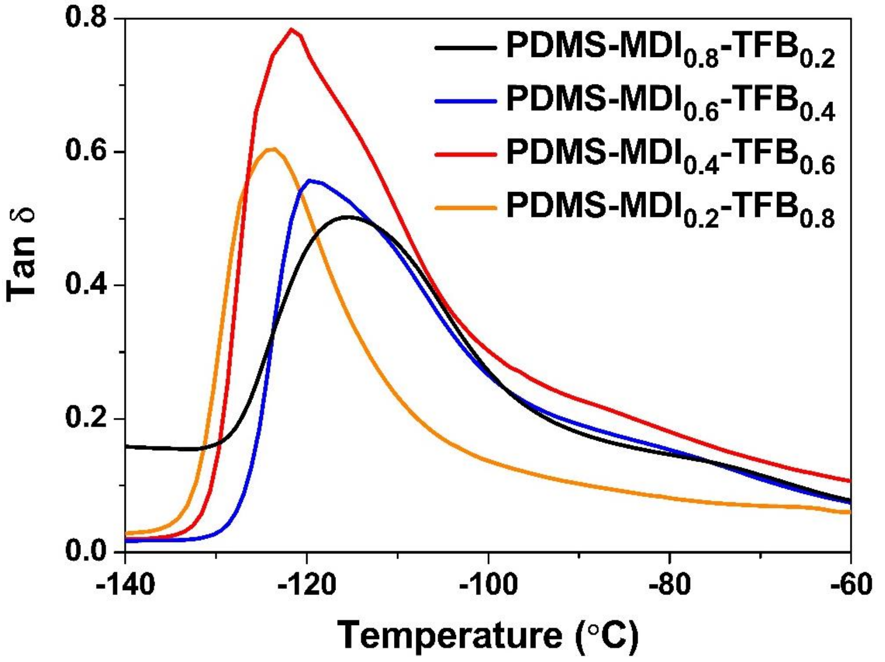

Figure S5. DMA curves of PDMS-MDI$_x$-TFB$_{1-x}$.

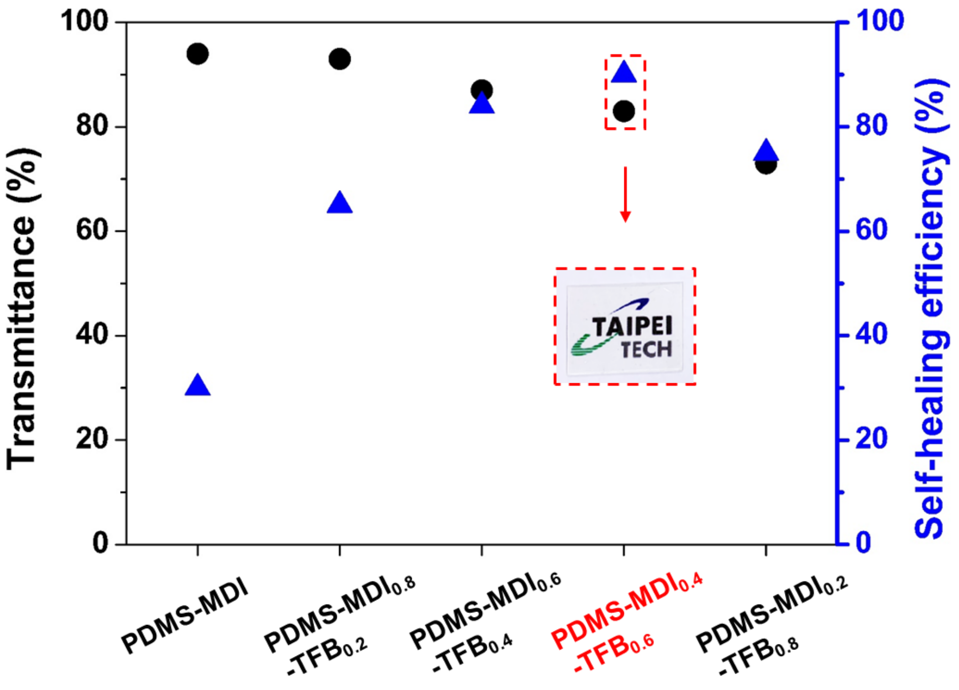

Figure S6. Optical transmittance curves of PDMS-MDI$_x$-TFB$_{1-x}$ with different rations as a function of self-healing efficiency. The inset represents the optically transparent image of PDMS-MDI$_{0.4}$-TFB$_{0.6}$ (3 cm x 2.5 cm).

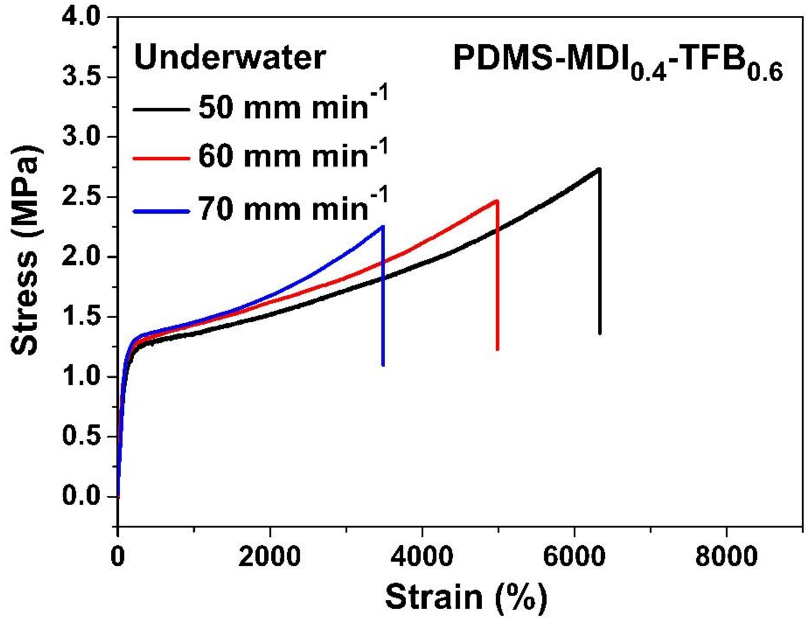

Figure S7. Stress–strain curves of the PDMS-MDI$_{0.4}$-TFB$_{0.6}$ film with different loading rate 50 to 70 mm min^{-1} after underwater healing process.

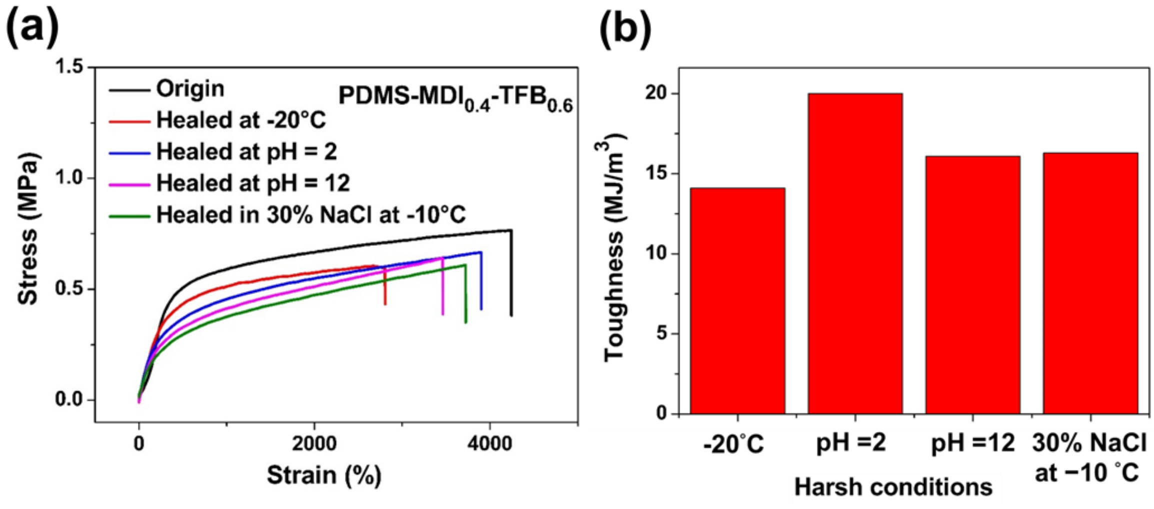

Figure S8. (a) The stress–strain curves of PDMS-MDI$_{0.4}$-TFB$_{0.6}$ film healed under various harsh conditions. (b) Toughness performances under various harsh conditions.

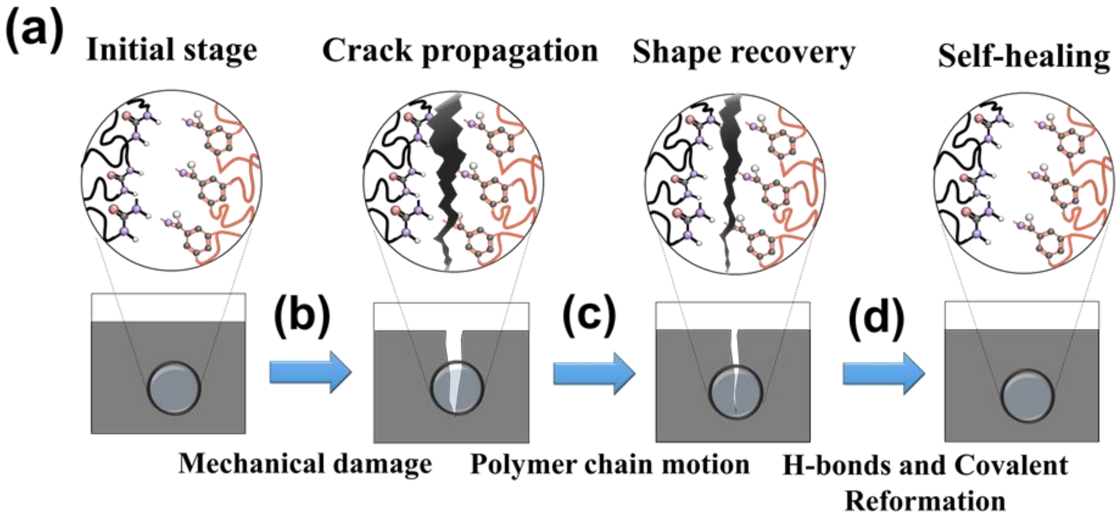

Figure S9. Schematic diagrams of room-temperature autonomous self-healing mechanism of PDMS-MDI_x-TFB_{1-x} elastomer film. a) Illustration of reversible imine bonds and dynamic hydrogen bonds dissociation–association under room-temperature autonomous self-healing process. b-d) Illustration of mechanical damaged induced chain conformational change, followed by chain motion within the damaged area and the reformation of H-bond and covalent imine bond upon self-healing film.

We seek to exploit the synergistic effect of imine bond to both dissipate energy and participate in the self-healing and H-bond array reconstruction for high toughness and elasticity. The autonomous self-healing mechanism relies on the retrieval of negative free energy resulting in a stable healed state and a metastable broken state (**Figure S9a**). To trigger the self-healing mechanism, the global free energy has to drive from a positive to a negative value to result in a stable thermodynamic state. First, upon mechanical damage, the polymeric chains at the damaged area undergo conformational entropic reduction caused by the polymeric segments stretching and compression within a locally reduced volume. The H-bond array destruction provides the enthalpic penalty in the form of heat to generate the condition responsible for spontaneous self-healing (**Figure S9b**). Second, upon imine and H-bond breaking, the strain energy dissipation will increase the internal energy of the system in the form of heat. As a result, freed end-chain from tethered or totally freed chains locally increases the enthalpy of the system at the cracked area. [35,36] To maintain the enthalpy penalty relatively high until complete self-healing, imine bond metathesis reaction allows diffusing locally energy within the reconstructing area (**Figure S9c**). Third, upon release of the mechanical constraint, our SHP flexibility guaranteed to recover the thermodynamically stable state that is the self-healed SHP through the increase of entropy from the stored conformational entropy. We speculated that at the damaged area, the stored conformational entropy is slowly released to

gradually recover autonomously the SHP entropy while sustaining stiffness and elasticity. The controlled release of the conformational entropy is the driving force to observe the slow and constant diffusing of broken chains within the cracked area. Imine bond as the stable and versatile cleaved bond will favor self-healing through favorable kinetic and thermodynamic network conditions; whereas the reconstruction of the cooperative H-bond array enables to decrease the enthalpic energy of the SHP, leading to a significant increase of the entropic contribution through the relaxation of the polymer chains within the previously cracked area (**Figure S9d**). Therefore, the resulting shifting of energetic contribution is the key to obtain a swift recovery of the damaged SHP state.

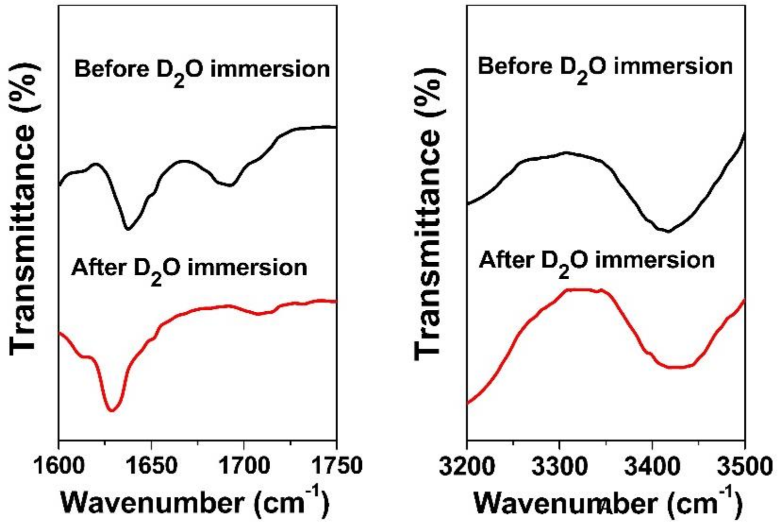

Figure S10. ATR-IR spectra of PDMS-MDI_x-TFB_{1-x} film before D_2O immersion (black line) and after D_2O immersion (red line); displaying variation of the −C=O and −NH of urea, C=N, and free aldehyde groups of TFB, respectively; Representing hydrogen/deuterium (H/D) exchange (interactions) between urea-based H-bonds/TFB-based imine/amine exchange and D_2O.

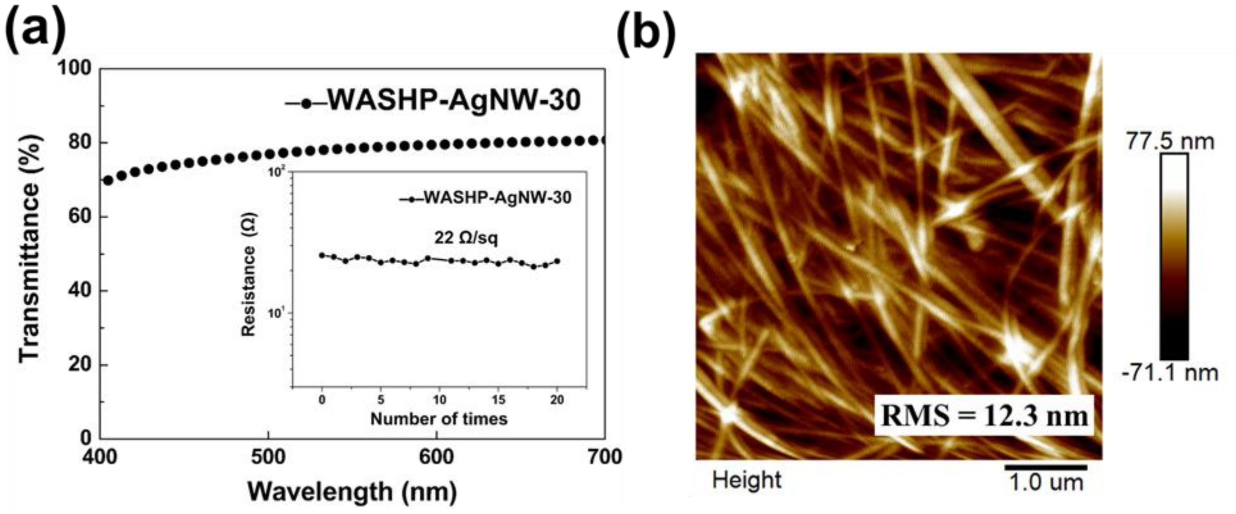

Figure S11. (a) Optical transmittance curves of PDMS-$MDI_{0.4}$-$TFB_{0.6}$ as a function of the wavelength. The inset show the average sheet resistance of PDMS-$MDI_{0.4}$-$TFB_{0.6}$. (b) Tapping mode AFM images of the WASHP-AgNW-30 electrode.

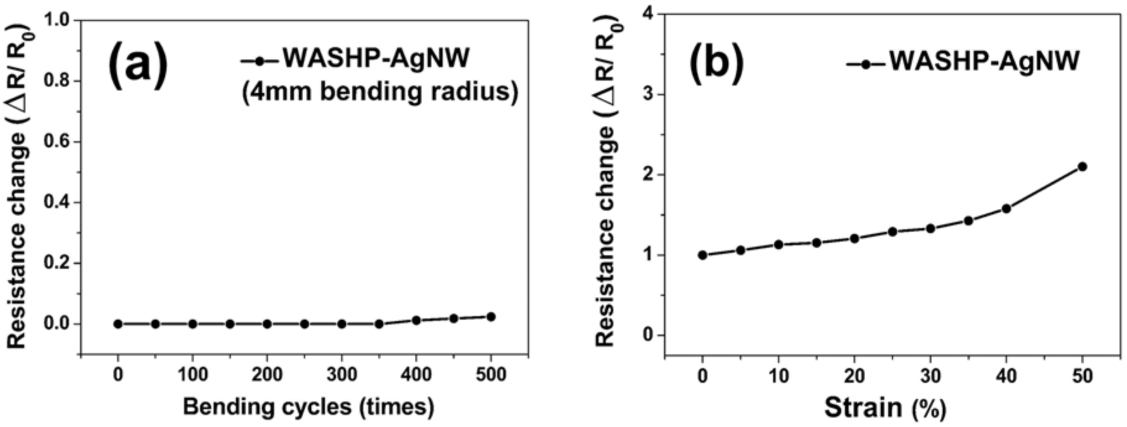

Figure S12. Relative change in the resistance of WASHP-AgNW electrodes with difference test (a) bending test (bending radius of 4 mm) and (b) strain test (from 0% to 50%).

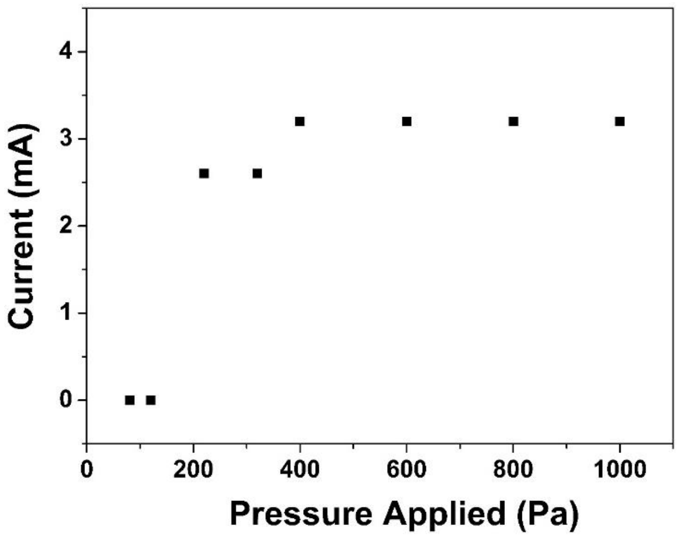

Figure S13. Transient current responses against low to high pressure ranges.

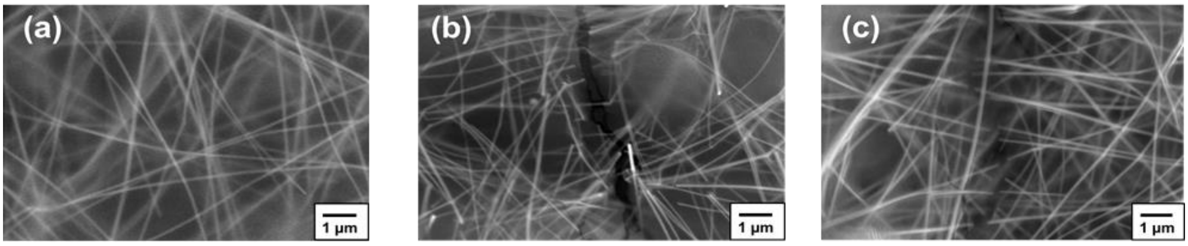

Figure S14. SEM morphology of WASHP-AgNW electrodes with various states (a) initial, (b) cut, and (c) self-healing.

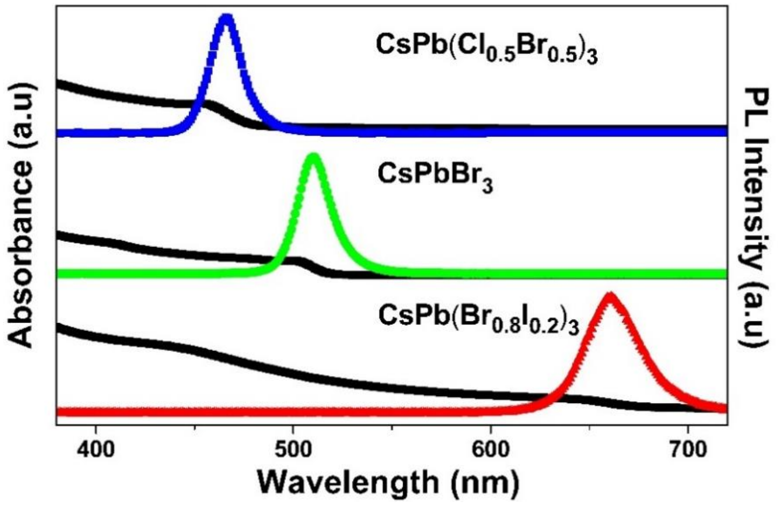

Figure S15. Optical absorption/PL spectra for $CsPb(Cl_{0.5}Br_{0.5})_3$ QDs (blue), $CsPbBr_3$ QDs (green), and $CsPb(Br_{0.8}I_{0.2})_3$ QDs (red), respectively.

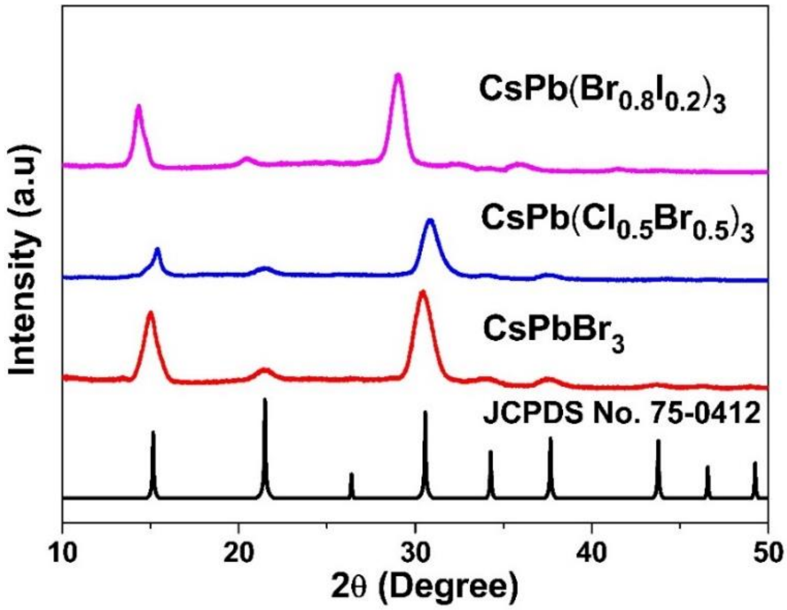

Figure S16. XRD patterns of $CsPb(Cl_{0.5}Br_{0.5})_3$ QDs (blue), $CsPbBr_3$ QDs (green), $CsPb(Br_{0.8}I_{0.2})_3$ QDs (red), and standard diffraction data of cubic $CsPbBr_3$ crystal (JPCDS No. 75-0412), respectively.

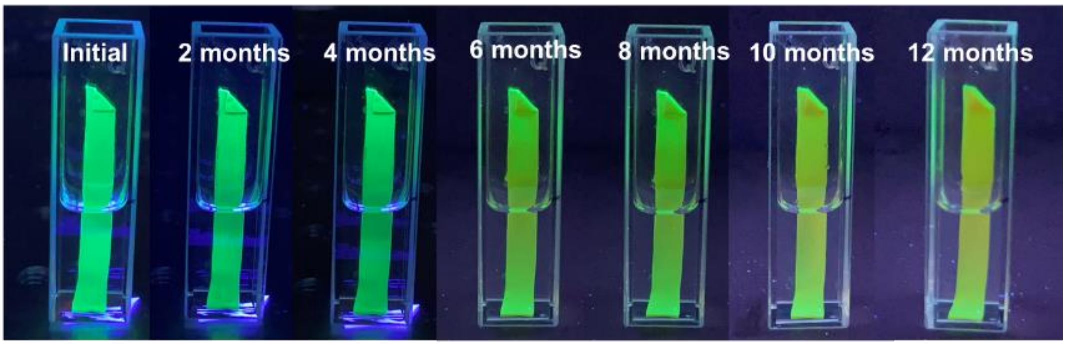

Figure S17. PL pictures of WASHP-QD (green) composites under UV-light (365nm) excitation upon immersed in boiling water.

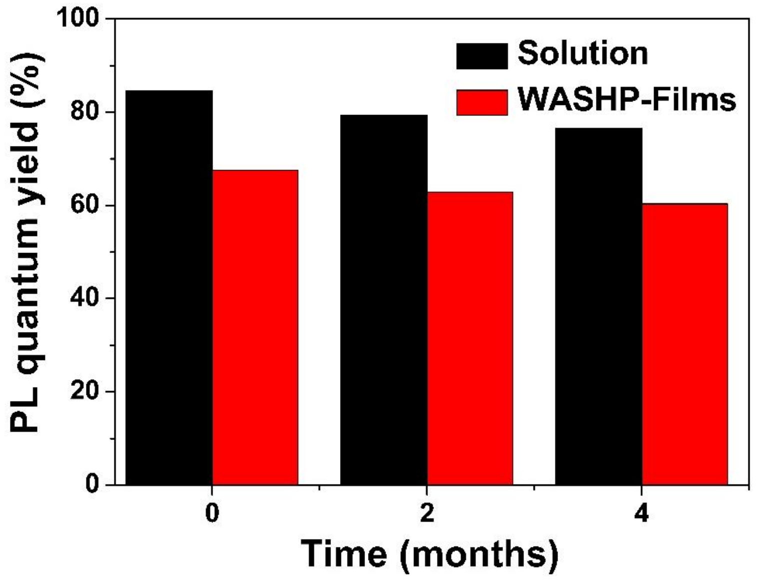

Figure S18. The quantum yield of WASHP-QD (green) composites.

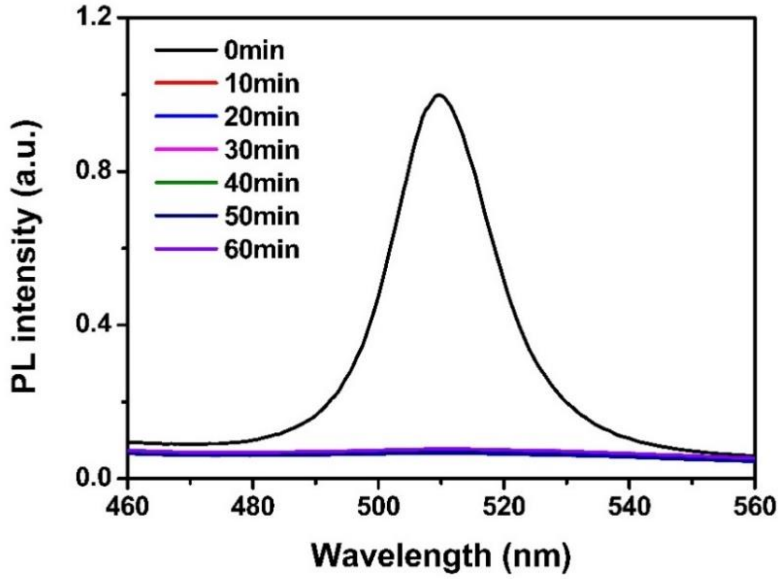

Figure S19. PL spectra of pristine perovskite QDs under boiled water medium.

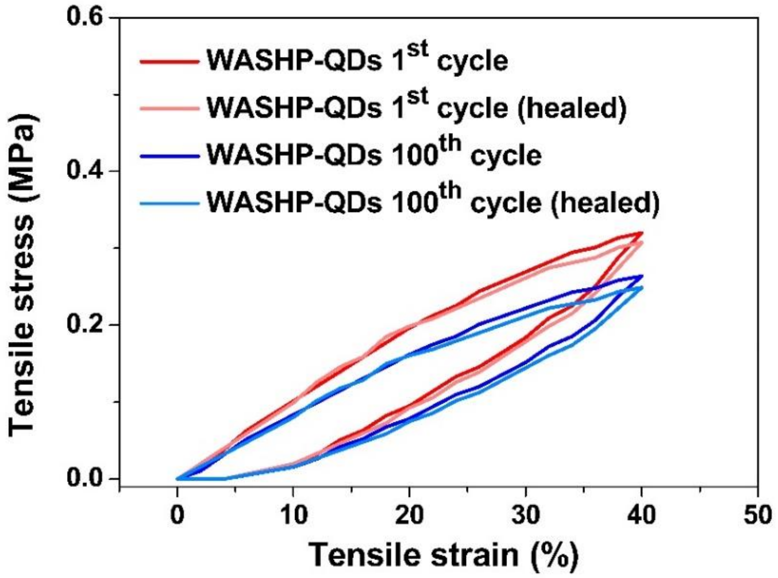

Figure S20. The stress–strain curve of WASHP-QD composites in different healing condition in cyclic stress–strain tests (40% strain).

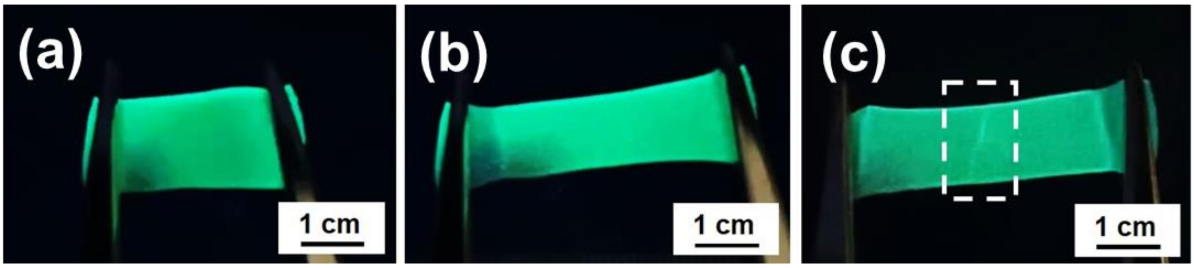

Figure S21. Photoluminescent pictures of (a) Initial WASHP-QD composites (b) WASHP-QD composites on stretching 100% (c) Healed WASHP-QD composites on stretching 100%.

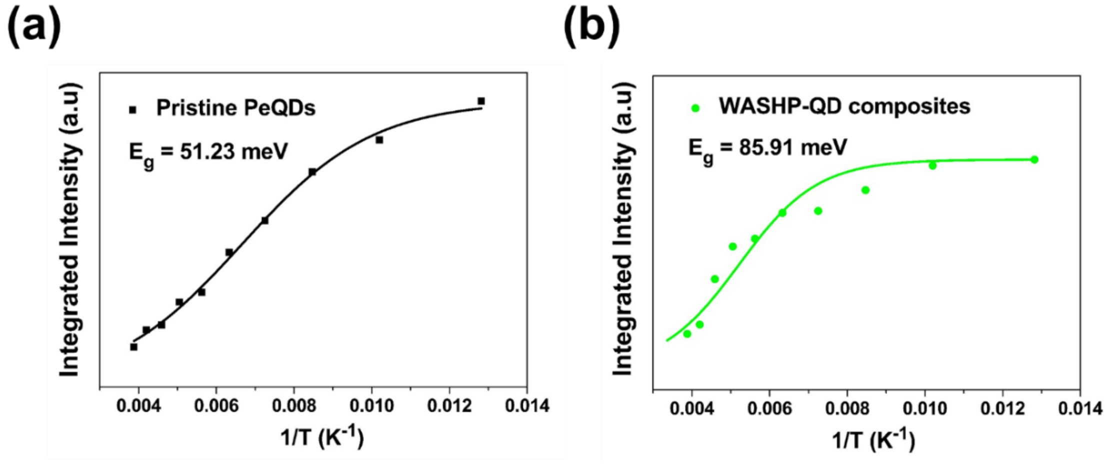

Figure S22. Temperature-dependent PL spectra of (a) pristine PeQDs and (b) WASHP-QD composites.

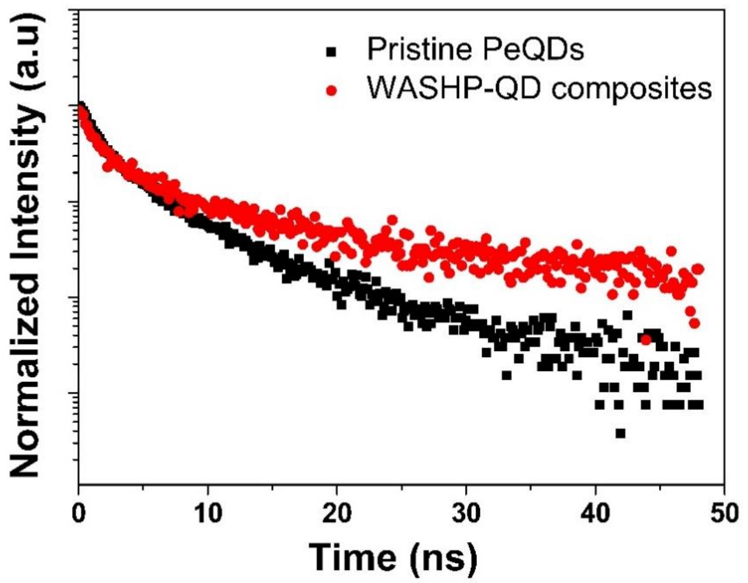

Figure S23. TRPL decay curves of pristine PeQDs and WASHP-QD composites.

Table S1. Molecular and mechanical characteristics of PDMS-MDI_x-TFB_{1-x}.

Sample	M_n	PDI	T_g ℃)	Elongation (%)	Young's modulus (MPa)	Toughness (MJ/M^3)	Self-Healing efficiency (%)
**PDMS-MDI**	47498	1.64	-	600	0.86	8.2	30
**PDMS-$MDI_{0.8}$-$TFB_{0.2}$**	41393	1.50	-115	1788	0.45	15.7	65
**PDMS-$MDI_{0.6}$-$TFB_{0.4}$**	37242	1.57	-119	2715	0.21	21.2	84
**PDMS-$MDI_{0.4}$-$TFB_{0.6}$**	36648	1.49	-121	4250	0.18	26.8	90
**PDMS-$MDI_{0.2}$-$TFB_{0.8}$**	20921	1.45	-123	5300	0.15	19.5	75
**PDMS-TFB**	11937	1.55	-	2570	NA	NA	NA

Table S2. Comparison of this study and previous perovskite composites and its emission stability evolution towards air, moisture, and water.

Materials	PL retained	Condition	Stability	References
$MAPbBr_3$ NC@2P	96%	Ambient conditions	1 month	1
PMMA sealed perovskite NWs	74%	80% RH	1 month	2
DA capped $FAPbBr_3$ QDs	~100%	60 ℃ and 90% RH	1000 hours	3
Mn doped perovskite-silica gel	80%	90% RH	1 month	4
BCP-$MAPbBr_3$	~95%	70% RH	50 hours	5
CPBr-NB-AE	40%	Water	3.5 hours	6
SR/PVP-$CsPbBr_3$	-	Water	4 hours	7
Perovskite@MOG	78%	Water	12 hours	8
$CsPbBr_3$-PSZ	30%	Water	150 hours	9
PMAO coated $CsPbBr_3$ QDs	60%	Water	24 hours	10
$CsPbBr_3$@PDEP	33%	Water	30 days	11
$CsPbBr_3$@CA-SiO_2	59%	Water	8 days	12
$CsPbBr_3$@SBS fiber	80%	Water	90 days	13
CsPbBr3/PBA-co-PNMA	32%	Water	6 hours	14
1D, 2D, 3D-$CsPbBr_3$ QDs	~99%	Water	30 days	15
**WASHP-$CsPbBr_3$ QDs**	**84%**	**Boiled Water**	**12 months**	**This Work**

Table S3. Physical parameters of Pb and the estimated A_e values.

Sample	D (cm/s)	C_s (kg/m^2)	C_b (kg/m^2)	d (cm)	dm/dt (kg/s)	A_e (m^2)
Pristine-PeQDs-film	9.45 x10^{-6}	1.35 x10^{-3}	0	0.5	5.55 x10^{-11}	2.18 x10^{-3}
WASHP-PeQDs-fresh	9.45 x10^{-6}	1.35 x10^{-3}	0	0.5	6.77 x10^{-16}	2.65 x10^{-8}
WASHP-PeQDs-healed	9.45 x10^{-6}	1.35 x10^{-3}	0	0.5	8.32 x10^{-16}	3.26 x10^{-8}

The Pb concentration of pristine QDs films were diluted and calculated approximately 100 ppm after using an ICP-MS instrument. We have investigated the Pb leakage rate of our WASHP-QDs composite before and after healing at room temperature based on previous research methods[16,17] . The following Noyes-Whitney equation have been used to study the metal leakage from perovskite.

$$\frac{dm}{dt} = A_e\{\frac{D}{d}\}(C_s - C_b)$$

where m is the mass of the dissolved Pb, t is the diffusion time, D is the diffusion coefficient of Pb^{2+} ions, d is the thickness of the self-healing polymer layer, C_s is the saturated mass concentration at the surface of the solution, and C_b is the mass concentration in the bulk solution. A_e is the surface area of the solution (m^2). The value of parameters for (D, C_s and C_b) are calculated from previous literature [16,17] and (d and dm/dt) are calculated by our experimental results.

Table S4. TRPL lifetime comparison of pristine PeQDs

and WASHP-QD composites.

Sample	τ_1 (ns)	A1 (%)	τ_2 (ns)	A2 (%)	τ_{av} (ns)
Pristine PeQDs	5.68	0.32	1.25	0.75	4.17
WASHP-QD composites	8.56	0.18	1.82	0.92	5.05

References

[1] L.-J. Xu, M. Worku, H. Lin, Z. Xu, Q. He, C. Zhou, H. Zhang, Y. Xin, S. Lteif, J. Xue, B. Ma, *J. Phys. Chem. Lett.* **2019**, *10*, 5923.

[2] C.-H. Lin, T.-Y. Li, J. Zhang, Z.-Y. Chiao, P.-C. Wei, H.-C. Fu, L. Hu, M.-J. Yu, G. H. Ahmed, X. Guan, C.-H. Ho, T. Wu, B. S. Ooi, O. F. Mohammed, Y.-J. Lu, X. Fang, J.-H. He, *Nano Energy* **2020**, *73*, 104801.

[3] J. Tong, J. Luo, L. Shi, J. Wu, L. Xu, J. Song, P. Wang, H. Li, Z. Deng, *J. Mater. Chem. A* **2019**, *7*, 4872.

[4] S. Wang, D. Wu, S. Yang, H. Zhen, Z. Lin, Q. Ling, *J. Mater. Chem. C* **2020**, *8*, 12623.

[5] H. Han, B. Jeong, T. H. Park, W. Cha, S. M. Cho, Y. Kim, H. H. Kim, D. Kim, D. Y. Ryu, W. K. Choi, C. Park, *Adv. Funct. Mater.* **2019**, *29*, 1808193.

[6] S. Lou, T. Xuan, C. Yu, M. Cao, C. Xia, J. Wang, H. Li, *J. Mater. Chem. C* **2017**, *5*, 7431.

[7] J. Hai, H. Li, Y. Zhao, F. Chen, Y. Peng, B. Wang, *Chem. Commun.* **2017**, *53*, 5400.

[8] S. Mollick, T. N. Mandal, A. Jana, S. Fajal, S. K. Ghosh, *Chem. Sci.* **2019**, *10*, 10524.

[9] H. C. Yoon, Y. R. Do, *ACS Appl. Mater. Interfaces* **2019**, *11*, 22510.

[10] H. Wu, S. Wang, F. Cao, J. Zhou, Q. Wu, H. Wang, X. Li, L. Yin, X. Yang, *Chem. Mater.* **2019**, *31*, 1936.

[11] W. Yang, L. Fei, F. Gao, W. Liu, H. Xu, L. Yang, Y. Liu, *Chem. Eng. J.* **2020**, *387*, 124180.

[12] A. Pan, Y. Li, Y. Wu, K. Yan, M. J. Jurow, Y. Liu, L. He, *Mater. Chem. Front.* **2019**, *3*, 414.

[13] D.-H. Jiang, Y.-H. Tsai, L. Veeramuthu, F.-C. Liang, L.-C. Chen, C. C. Lin, T. Satoh, S.-H. Tung, C.-C. Kuo, *APL Materials* **2019**, *7*, 111105.

[14]L. Laysandra, Y. J. Fan, C. Adena, Y.-T. Lee, A.-N. A. Duong, L.-Y. Chen, Y.-C. Chiu, *Front. Chem.* **2020**, *8*, 766.

[15] A. Pramanik, S. Patibandla, Y. Gao, K. Gates, P. C. Ray, *JACS Au.* **2021**, *1*, 53.

[16] Y. Jiang, L. Qiu, E.J. Juarez-Perez, L. L. K. Ono, Z. Hu, Z. Liu, Z. Wu, L. Meng, Q. Wang, Y. Qi, *Nat Energy* **2019**, *4*, 585.

[17] I, Celik, Z, Song, A.B. Phillips, M.J. Heben b, D. Apul, *J. Clean. Prod.* **2018**, *186*, 632.
